# Expanding the Diversity of Plant Monoterpenoid Indole Alkaloids Employing Human Cytochrome P450 3A4

**DOI:** 10.1002/cbic.202000020

**Published:** 2020-04-06

**Authors:** Yuriy V. Sheludko, Jascha Volk, Wolfgang Brandt, Heribert Warzecha

**Affiliations:** ^1^ Plant Biotechnology and Metabolic Engineering Technische Universität Darmstadt Schnittspahnstraße 3–5 64285 Darmstadt Germany; ^2^ Leibniz Institute of Plant Biochemistry Weinberg 3 06120 Halle/Saale Germany

**Keywords:** natural products, cytochromes, monoterpenoid indole alkaloids, CYP3A4, vinorine

## Abstract

Human drug‐metabolizing cytochrome P450 monooxygenases (CYPs) have enormous substrate promiscuity; this makes them promising tools for the expansion of natural product diversity. Here, we used CYP3A4 for the targeted diversification of a plant biosynthetic route leading to monoterpenoid indole alkaloids. In silico, in vitro and in planta studies proved that CYP3A4 was able to convert the indole alkaloid vinorine into vomilenine, the former being one of the central intermediates in the ajmaline pathway in the medicinal plant *Rauvolfia serpentina* (L.) Benth. ex Kurz. However, to a much larger extent, the investigated conversion yielded vinorine (19*R*,20R)‐epoxide, a new metabolite with an epoxide functional group that is rare for indole alkaloids. The described work represents a successful example of combinatorial biosynthesis towards an increase in biodiversity of natural metabolites. Moreover, characterisation of the products of the in vitro and in planta transformation of potential pharmaceuticals with human CYPs might be indicative of the route of their conversion in the human organism.

## Graphical abstract

**Alternative oxidation**: We used CYP3A4 for the targeted diversification of plant monoterpenoid indole alkaloids. In vitro and in planta studies proved that the cytochrome mainly converted vinorine into vinorine (19*R*,20*R*)‐epoxide, a new metabolite containing a rare for indole alkaloid epoxide group. A minor route led to the formation of the natural constituent vomilenine.

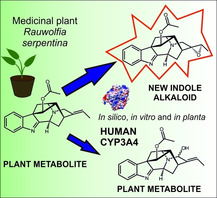

Cytochrome P450 monooxygenases (CYPs) are versatile enzyme catalysts, capable of performing a variety of reactions, most of which are oxidation reactions.^1^ CYPs are very often key components of both anabolic and catabolic reactions in organisms of all kingdoms of life. In humans, roughly eight of the total of 57 CYPs are responsible for the metabolism of most xenobiotics entering the body via food or as medicines. Especially the human CYP isotype 3A4 has tremendous substrate promiscuity, enabling it to act on roughly half of all ingested pharmaceuticals and making it a key player in the metabolism and pharmacodynamics of numerous important medicines.^2^ Due to its versatility, CYP3A4 became a promising tool in chemical and biotechnological applications,^3^, ^4^ affording regiospecific modulation of target molecules, which is hardly achievable via chemical modification.^5^

Biosynthetic pathways leading to the formation of complex plant specialized metabolites very often encompass numerous CYP enzymes. The importance of these catalysts for a given plant's metabolism is underscored by the number of genes corresponding to this enzyme family, ranging from around 150 to more than 400, depending on the plant species.^6^ In the biosynthetic route leading to the build‐up of the antiarrhythmic drug ajmaline in the medicinal plant *Rauvolfia serpentina* (L.) Benth. ex Kurz., at least seven different CYP enzymes are involved.^7^, ^8^ In many cases, as partly determined in the closely related plant species *Catharanthus roseus*, they perform very distinguished reactions, like regio‐ and stereospecific hydroxylation of 7‐deoxyloganic acid to form loganic acid (CYP72A224), decyclization of loganin (CYP72A1) or the multi‐step oxidation of nepetalactol (CYP76A26) 7 (Figure S1). However, some CYPs, like the recently discovered CYP82S18 (vinorine hydroxylase, VH from *R. serpentina* 9), diversify the biosynthetic route into at least two branches. By hydroxylating the central intermediate vinorine at C21 (Figure 1), VH forms vomilenine, which can subsequently be converted to ajmaline. The second reaction performed by the CYP is the non‐oxidative isomerization of vomilenine to perakine. In the course of this reaction enzymatically induced ring‐opening leads to the rotation around the C15‐C20 bond and Michael addition of an amine to the α,β‐unsaturated aldehyde followed by further irreversible tautomerization of the formed enol to the aldehyde.^9^ Formation of perakine opens a side‐path of this metabolic network toward the alkaloid raucaffrinoline and (19*S*,20*R*)‐19,20‐dihydroperaksines, the latter of which feature a different structural scaffold.^8^

**Figure 1:**
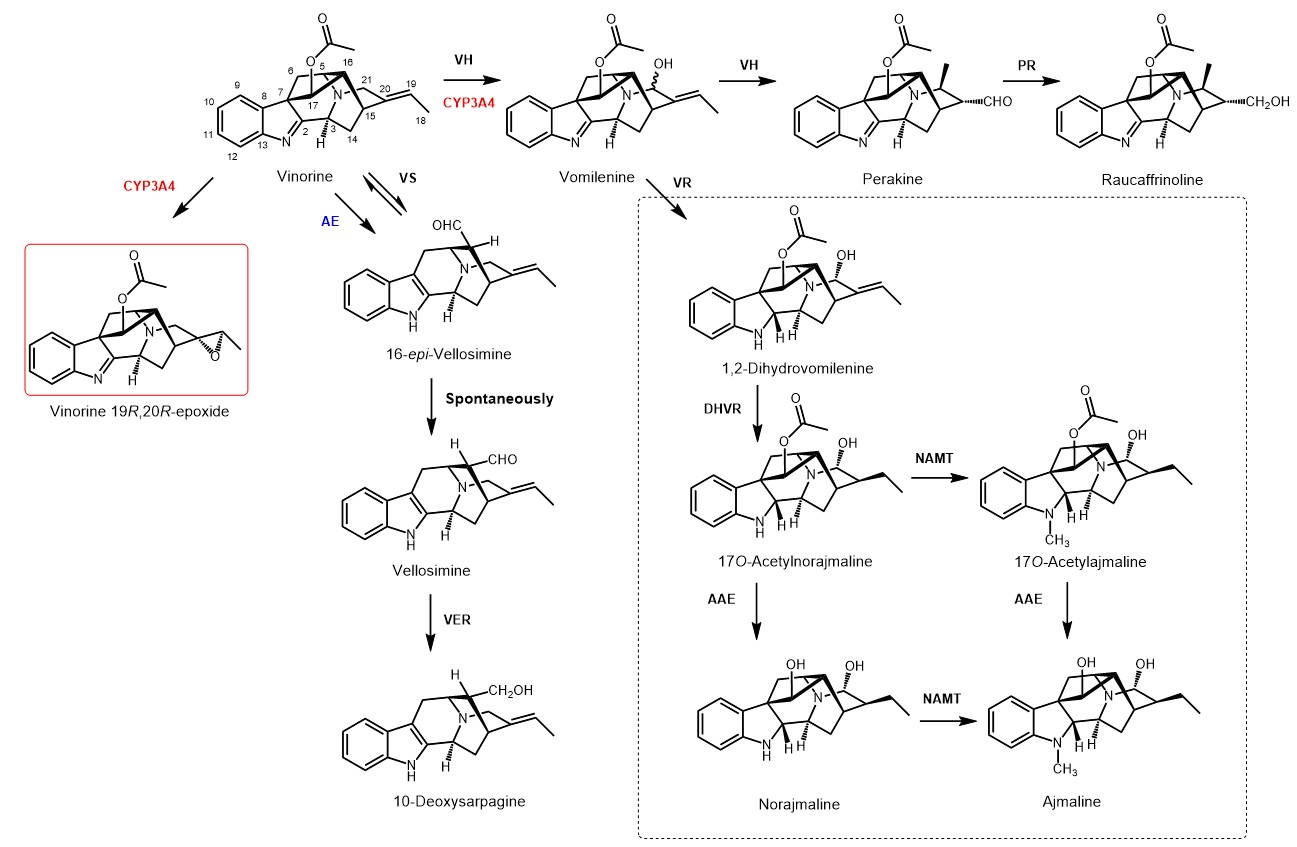
Selected routes of enzymatic conversion of vinorine as an important intermediate in the ajmaline biosynthesis pathway. Enzymes that catalyse reactions in *R. serpentina* are indicated in black: acetylajmaline esterase (AAE), 1,2β‐dihydrovomilenine reductase (DHVR), norajmaline Nα‐methyltransferase (NAMT), perakine reductase (PR), vellosimine reductase (VER), vinorine hydroxylase (VH), vinorine synthase (VS), vomilenine reductase (VR); human CYP3A4 is indicated in red; the putative unspecific acetyl esterase (AE) from the baculosome preparation is indicated in blue. The new alkaloid vinorine (19*R*,20*R*)‐epoxide is shown in a red frame. The indoline alkaloids of the late stages of the ajmaline pathway are shown in a black dashed frame.

The indolenine alkaloid vinorine, one of the central intermediates in the pathway, was recently shown to facilitate regeneration and to promote recovery of motor function after nerve crush injury via up‐regulation of nerve growth factor protein levels and extracellular signal‐regulated kinase activation.^10^ This finding drives particular interest in the elucidation of the biosynthetic routes and the generation of novel and potentially pharmaceutically significant derivatives of this alkaloid. Moreover, although the group of known indole alkaloids comprises more than 3000 members, little is known about their metabolism by liver CYPs: the SuperCYP database contains only a limited number of records on the conversion of these xenobiotics.^11^ CYP‐mediated oxidation may lead not only to xenobiotic detoxification but also to the modification of its biological activity and/or prolongation of the half‐life.^12^, ^13^ In this concern, characterisation of the products of in vitro or in planta human CYP reaction with potential pharmaceuticals might be helpful for a better understanding of their transformation in the human organism.

To investigate regio‐ and stereospecificity of an alternative CYP enzyme for the oxidation of vinorine, we aimed at probing a human drug‐metabolizing CYP for its suitability to substitute the endogenous VH from *R. serpentina*. We assayed whether this CYP can provide a more specific vinorine hydroxylating activity excluding formation of the side product perakine or, alternatively, produce recently unknown metabolites. Since previous work on human CYP2D6 transiently expressed in *Nicotiana benthamiana* did not show any vinorine conversion,^14^ we decided to explore the capabilities of CYP3A4, which has been reported to accept a greater variety of substrates than CYP2D6. Structural features enabling promiscuity of the enzyme have been investigated in detail.^15^

To evaluate whether vinorine could be a possible substrate for CYP3A4, we performed docking studies using the MOE 2019.01 software (S1.6). One hundred docking positions of vinorine to the active site were generated in proximity to the haem group of the CYP. Out of 20 best‐scored alternative docking positions, the second best‐scored featured a small distance between the double bond of vinorine and the hydroperoxo‐ferric complex in the active centre. To check the stability of the arrangement, a final energy minimization with the fixed backbone atoms was performed. The resulting tertiary enzyme structure with the docked vinorine is displayed in Figure 2A, while the detailed depictions of its active site are featured in Figure 2B and C.

**Figure 2:**
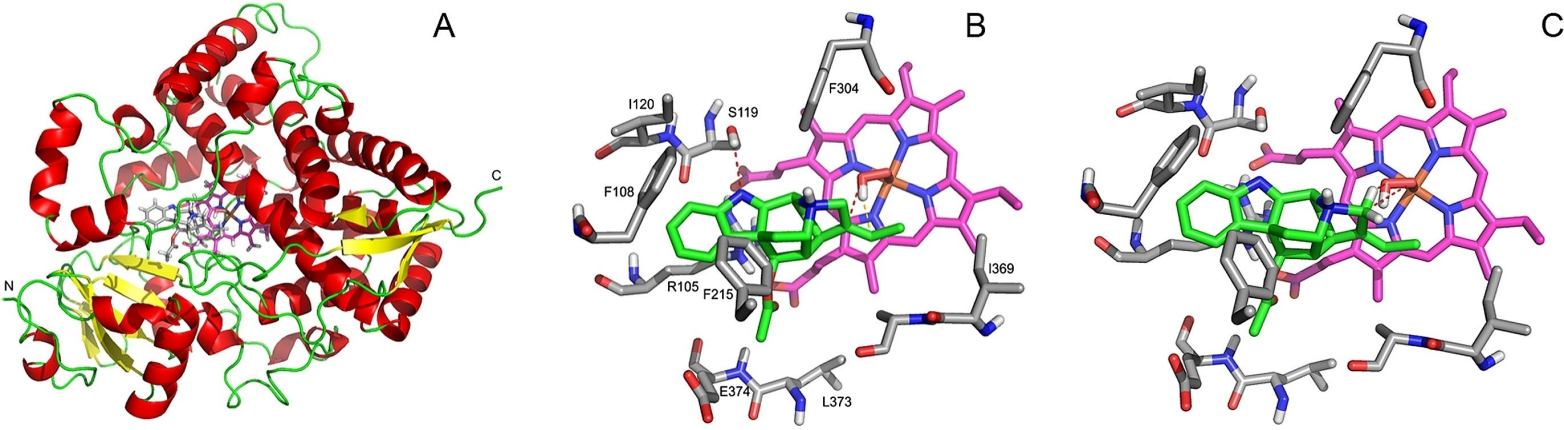
3D Model and active site of CYP3A4 (PDB ID, 1TQN). A) Tertiary structure of CYP3A4 with docked vinorine (grey sticks); haem (magenta C atoms) located in the centre of the figure. B) Active site with bound vinorine (green C atoms); the formation of a hydrogen bond of the NH group of the indene moiety with S119 is indicated by a dashed line; the red and yellow dashed lines indicate the reaction to form the epoxide (vinorine (19*R*,20*R*)‐epoxide). C) Docking pose as in B, with red dashed lines indicating the reactions to form vomilenine.

The postulated docking arrangement is mainly recognised by the hydrophobic amino acid residues F215, F108 and F304 and accompanied by the formation of a hydrogen bond of the NH group of the indene moiety with S119. With this docking pose, the C19−C20 double bond of the substrate is in very close proximity (3.03 Å) to the oxygen atom of the ferric hydroperoxo intermediate, which allows effective generation of the epoxide (vinorine 19,20‐epoxide; Figure 2B). Further, the distance between the distal oxygen atom of the hydroperoxo‐ferric haem intermediate and equatorial hydrogen atom at C21 in the vinorine molecule is very small (2.3 Å; Figure 2C). After activation, the proximal oxygen atom in this complex can attack the C21 carbon atom (distance, 4.07 Å, second red dashed line in Figure 2C) to form vomilenine. To prove the modelling data experimentally, vinorine was isolated from hairy roots of *R. serpentina* (purity, 91 %; perakine content, ∼5–7 %), as described earlier,^16^ and its identity was verified by comparison with the authentic reference sample (HPLC‐ESI‐MS) and by NMR analysis (Figures S2–S7). The isolated alkaloid was used as a substrate in the subsequent enzyme assays.

Two experimental systems were explored for the oxidation of vinorine by CYP3A4: a) in vitro assays with baculosome preparations of recombinant CYP3A4 (S1.2) and b) in planta transient system expressing CYP3A4^14^ (S1.4).

The HPLC‐ESI‐MS chromatograms of baculosome assay samples after 4 h of incubation displayed a major new peak at 8.8 min (peak 1, *m/z* [M+H]^+^ 351) and a minor peak at 9.4 min (peak 2, *m/z* [M+H]^+^ 351) as compared to the controls, together with a distorted peak characteristic of an indole alkaloid with an aldehyde functional group (Figure S8). After the addition of sodium borohydride, the distorted peak disappeared and an alkaloid with *t*_R_ **=**8.5 min and *m/z* [M+H]^+^ 295 was registered as a major component of the reaction mixture (Figure 3A; peak 2). This compound was identified as 10‐deoxysarpagine by comparison with the authentic reference sample (53 % yield). 10‐Deoxysarpagine is the reduced form of the known sarpagine alkaloid vellosimine, containing an aldehyde group (Figure 1). Vellosimine was also formed as a major product (66 % yield, calculated based on 10‐deoxysarpagine content after reduction) in the control sample lacking NADPH but supplemented with vinorine and CYP3A4 (Figure 3B; peak 2). In the samples where enzymes were inactivated by boiling, the detected formation of this alkaloid was considerably lower (13 % yield, calculated based on 10‐deoxysarpagine content after reduction; Figure 3C; peak 2). We propose that the generation of vellosimine was triggered by an unspecific esterase from the baculosome preparation or, to a lesser extent, is the result of vinorine deacetylation during the borohydride reduction (as observed in heat‐inactivated samples). Removal of the acetyl residue causes destabilization of the indolenine skeleton followed by cleavage of the C7−C17 bond and formation of the sarpagane‐type alkaloid 16‐*epi*‐vellosimine, which undergoes spontaneous irreversible epimerisation into vellosimine.^8^

**Figure 3:**
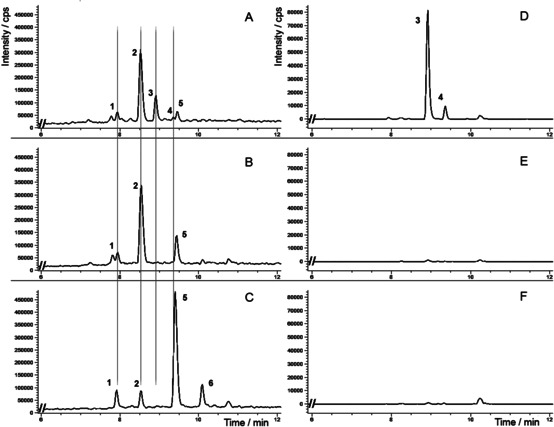
Fragments of HPLC‐ESI‐MS ion chromatograms of CYP3A4 baculosome samples after sodium borohydride reduction. A) CYP3A4 baculosome preparation, vinorine and NADPH; B) CYP3A4 baculosome preparation, vinorine, lacking NADPH (control 1); C) heat‐inactivated CYP3A4 baculosome preparation, vinorine and NADPH (control 2); D)–F) respective selective extraction of *m/z* 351 ion chromatogram. 1, raucaffrinoline (*t*_R_ **=**7.9 min, *m/z* [M+H]^+^ 353); 2, 10‐deoxysarpagine (*t*_R_ **=**8.5 min, *m/z* [M+H]^+^ 295); 3, vinorine (19*R*,20*R*)‐epoxide (*t*_R_ **=**8.8 min, *m/z* [M+H]^+^ 351); 4, vomilenine (*t*_R_ **=**9.4 min, *m/z* [M+H]^+^ 351); 5, vinorine (*t*_R_ **=**9.5 min, *m/z* [M+H]^+^ 335); 6, side‐product of vinorine after sodium borohydride reduction (*t*_R_ **=**10.1 min, *m/z* [M+H]^+^ 337).

Vellosimine was isolated after preparative conversion of vinorine by the CYP3A4 baculosome reagent, and its structure was confirmed by NMR^17^ (Figure S9 and S10).

A trace amount of raucaffrinoline (*t*_R_ **=**7.9 min, *m/z* [M+H]^+^ 353), detected in both control and experimental samples (6.0 % and 5.5 % of total ion current, respectively) after borohydride reduction, derived from perakine – the contaminant of the vinorine sample.

Therefore, conversion of vinorine by CYP3A4 led to the formation of a new major compound (*t*_R_ **=**8.8 min, 16 % yield) with *m/z* [M+H]^+^ 351 (Figure 3A; peak 3), and UV spectrum similar to that of vinorine (Figure S11), suggesting that the oxidation occurred outside of the UV‐absorbing chromophore. The novel alkaloid was extracted from the reaction mixture, purified by preparative TLC and its structure was elucidated by NMR (S1.3). The ^1^H NMR spectrum of the new alkaloid exhibited a profile of signals similar to vinorine within the indolenine part of the molecule, but was significantly divergent in the regions adjacent to C19−20 (Table 1, Figures S12–S18). The signal of H19 vinyl proton (δ 5.3 ppm, q, *J*=6.9 Hz) shifted upfield to δ 3.06 ppm (q, *J*=5.5 Hz), as did the signals of the protons at C15, C18 and C21, adjacent to the vinyl moiety in vinorine. This indicates the dislocation of the deshielding effect of the 19–20 double bond in the novel compound. The data of a CLIP‐COSY experiment displayed coupling of the H19 proton with the H18 methyl protons only and that of the H21 protons between themselves, suggesting a quaternary carbon at C20, which was subsequently confirmed by ^13^C NMR experiments (Table 1, Figure S16). The signals corresponding to C19 and C20 of the vinyl carbons in vinorine molecule (δ 116.04 ppm and δ 137.67 ppm) were detected at δ 62.34 ppm and δ 62.89 ppm, respectively, in the new compound. These positions are in good agreement with the chemical shifts of sp^3^ carbons of an oxirane ring, characteristic of, for instance, the known indole alkaloid tabernaricatine A.^18^

**Table 1:** ^1^H and ^13^C NMR data of vinorine (19*R*,20*R*)‐epoxide and vinorine in CDCl_3_. Values are in ppm. The multiplicities and coupling constants (*J* in Hz) are in parentheses. The equatorial (eq) and axial (ax) positions of protons are marked.

Position	Vinorine (19*R*,20*R*)‐epoxide		Vinorine	
	^1^H NMR	^13^C NMR	^1^H NMR	^13^C NMR
2	–	183.14^[a]^	–	183.59
3	4.29 (d, *J*=9.3, 1H)	55.85	4.24 (dd, *J*=7.5, 3.3, 1H)	56.26
5	3.53 (m, 1H)	58.28	3.42 (m, 1H)	58.22
6	(eq) 1.71 (d, J=11.9, 1H) (ax) 2.78 (dd, J=11.9, 4.8, 1H)	37.72	(eq) 1.71 (d, J=11.9, 1H) (ax) 2.75 (dd, J=11.9, 4.8, 1H)	37.34
7	–	64.10	–	64.52
8	–	136.37^[a]^	–	136.47
9	7.47 (d, *J*=7.6, 1H)	123.98	7.46 (d, *J*=7.6, 1H)	123.95
10	7.23 (td, *J*=7.5, 0.9, 1H)	125.73	7.22 (td, *J*=7.5, 1.1, 1H)	125.65
11	7.41 (td, *J*=7.6, 1.2, 1H)	128.96	7.39 (td, *J*=7.6, 1.2, 1H)	128.84
12	7.63 (d, *J*=7.6, 1H)	121.26	7.62 (d, *J*=7.6, 1H)	121.18
13	–	156.68^[a]^	–	156.68
14	(eq) 1.88 (dd, *J*=14.7, 5.1, 1H) (ax) 2.33 (dd, *J*=14.7, 9.3, 1H)	23.84	1.97–1.91 (m, 2H)	26.57
15	2.24 (m, 1H)	29.22	3.28 (m, 1H)	27.60
16	2.53 (t, *J*=6.1, 1H)	46.35	2.44 (td, *J*=6.3, 0.8, 1H)	49.09
17	4.99 (d, *J*=0.9, 1H)	77.77	5.03 (d, *J*=0.8, 1H)	77.84
18	1.41 (d, *J*=5.5, 3H)	14.41	1.67 (d, *J*=6.9, 3H)	13.07
19	3.06 (q, *J*=5.5, 1H)	62.34	5.32 (q, *J*=6.9, 1H)	116.04
20	–	62.89	–	137.67
21	(eq) 3.03 (d, *J*=14.5, 1H) (ax) 3.11 (d, *J*=14.5, 1H)	54.17	(eq) 3.53 (d, *J*=16.1, 1H) (ax) 3.57 (d, *J*=16.1, 1H)	54.18
CH_3_ (Ac)	2.18 (s, 3H)	21.27	2.17 (s, 3H)	21.28
C=O (Ac)	–	170.07^[a]^	–	170.08

[a] Quaternary carbon signal values were recorded in the ^13^C HMBC spectra.

To determine the stereochemistry of CYP3A4‐catalysed epoxidation, the data of the NOESY experiment were analysed (Figure S17). For H19, clear NOE interaction was observed only with the H18 protons of the methyl group; additionally, we registered strong signals between H18 protons and H15 as well as H18 and H16 (Figure S18). Interpretation of these data using 3D models of vinorine (*19*,*20*)‐epoxide led to the conclusion about the (19*R*,20*R*)‐configuration of the novel alkaloid (Figure S19A). The distance between the H18 protons and H16 in this configuration (≤3 Å) was appropriate for the induction of a clear NOE signal. In the possible alternative (19*S*,20*S*)‐configuration (Figure S19B), the distance between the mentioned protons increased to around 4 Å, while the distance between the H18 protons and the axial H14 (δ 2.33 ppm) was reduced to less than 3 Å. However, no through‐space interaction was detected between H18 and H14. Thus, oxidation of vinorine by CYP3A4 resulted in the formation of the novel product, vinorine (19*R*,20*R*)‐epoxide, containing an epoxide moiety, which is a rare feature for indole alkaloids.

The minor product of vinorine conversion in the CYP3A4 baculosome assay samples (Figure 3A, *t*_R_ **=**9.4 min; peak 4) was identified as vomilenine (2 % yield) upon comparison with the authentic reference sample (Figure S20). Selective extraction of the *m/z* 351 ion chromatogram did not allow detection of vomilenine in the control samples (Figure 3D–F; peak 4 and Figure S8D–F; peak 2). This confirms the possibility for alternative hydroxylation of the substrate in the CYP3A4 active centre, as predicted by our modelling (Figure 2C), leading to the formation of the known plant metabolite. Vomilenine is the major product of vinorine conversion by the plant CYP82S18,^9^ formed in the course of indole alkaloid biosynthesis.

Incubation of vomilenine with the CYP3A4 baculosome preparation did not result in its rearrangement into perakine (Figure S21), as described for the plant CYP82S18.^9^ Instead, we recorded signals with *m/z* [M+H]^+^ 367, which were products of vomilenine oxidation by CYP3A4 (Figure S22). Inefficient conversion (∼2 %, 9 % and 7 % yield) precluded structure elucidation of the corresponding alkaloids, but the similarity of their UV spectra to that of vomilenine (Figure S23) indicated that the oxidation occurred outside of the UV‐absorbing chromophore.

To evaluate if human CYP3A4 can be integrated into plant metabolic processes, we carried out transient expression of the CYP in *N. benthamiana* with subsequent administration of vinorine. This resulted in the formation of vinorine (19*R*,20*R*)‐epoxide, as confirmed by HPLC‐ESI‐MS analysis and comparison with the authentic reference sample (Figure S24). No other product of vinorine CYP3A4 oxidation was detected. The yield of the product was ∼4 % of the extracted alkaloids (as determined by HPLC and HPLC‐MS), probably due to the high activity of plant background enzymes competing with CYP3A4 for the substrate. As in the baculosome assays, the major component of both experimental and control plant samples was the product of vinorine deacetylation, vellosimine (Figure 1). It was partially reduced by plant background enzymes to 10‐deoxysarpagine, so that the yield of these two products exceeded 75 % of the extracted products.

In conclusion, we utilized CYP3A4 for the targeted diversification of the biosynthetic route of monoterpenoid indole alkaloids. In vitro and in planta studies proved that the human CYP mainly converted vinorine into vinorine (19*R*,20*R*)‐epoxide, a new metabolite, which was not previously found in nature or synthesized, containing an epoxide functional group that is rare for indole alkaloids. An alternative, minor route of vinorine hydroxylation by CYP3A4 led to the formation of vomilenine, which is the natural constituent of the plant indole alkaloid network. The described work represents a successful example of the realization of the combinatorial biosynthesis concept. This implies various combinations of heterologous enzymes and substrates in a different metabolic environment, to ultimately increase the biodiversity of natural metabolites. In addition, the characterisation of the products of the either in vitro or in planta transformation of compounds with potential pharmaceutical use with human CYPs might be indicative of the way of their conversion in the human organism.

## Conflict of interest

The authors declare no conflict of interest.

## Supporting information

As a service to our authors and readers, this journal provides supporting information supplied by the authors. Such materials are peer reviewed and may be re‐organized for online delivery, but are not copy‐edited or typeset. Technical support issues arising from supporting information (other than missing files) should be addressed to the authors.

Supplementary
