# Supplementary material for: Expanding the Diversity of Plant Monoterpenoid Indole Alkaloids Employing Human Cytochrome P450 3A4
Source: Chembiochem. 2020 Apr 6;21(14):1976–80. doi: 10.1002/cbic.202000020 (PMC7496586; doi:10.1002/cbic.202000020)
Supplement: Supplementary file 1 — Supplementary [file CBIC-21-1976-s001.pdf]

ChemBioChem

Supporting Information

Expanding the Diversity of Plant Monoterpenoid Indole Alkaloids Employing Human Cytochrome P450 3A4

Yuriy V. Sheludko,* Jascha Volk, Wolfgang Brandt, and Heribert Warzecha© 2020 The Authors. Published by Wiley-VCH Verlag GmbH & Co. KGaA. This is an open access article under the terms of the Creative Commons Attribution License, which permits use, distribution and reproduction in any medium, provided the original work is properly cited.

Supporting Information

TABLE OF CONTENS

**S1**	**Materials and Methods**	**4**
**S1.1**	**Genetic material, constructs and chemicals**	**4**
**S1.2**	**CYP3A4 baculosome assays**	**5**
**S1.3**	**Preparative extraction of vinorine 19*R*, 20*R*-epoxide**	**5**
**S1.4**	**Transient expression of *CYP3A4* in planta**	**5**
**S1.5**	**Analytical assays**	**7**
**S1.6**	**Computational design**	**7**
**S2.**	**Figures**	**8**
**Figure S1**	Selected routes of iridoid biosynthesis pathway	**8**
**Figure S2–S7.**	1D and 2D NMR spectra of vinorine in $CDCl_3$.	**9-14**
**Figure S8.**	Fragments of HPLC-MS ion chromatograms of CYP3A4 baculosome and control samples supplemented with vinorine.	**15**
**Figure S9–S10.**	1D NMR spectra of vellosimine in $CDCl_3$.	**16-17**
**Figure S11.**	HPLC-UV spectra of vinorine 19*R*, 20*R*-epoxide and vinorine.	**18**
**Figure S12–S18.**	1D and 2D NMR spectra of vinorine 19*R*, 20*R*-epoxide in $CDCl_3$.	**19-25**
**Figure S19.**	3D models of vinorine 19, 20-epoxide isomers.	**26**
**Figure S20.**	Fragments of HPLC-MS ion chromatograms of CYP3A4 baculosome samples supplemented with vinorine and standard vomilenine.	**27**
**Figure S21.**	Fragments of HPLC-MS ion chromatograms of CYP3A4 baculosome samples supplemented with vomilenine and standard perakine.	**28**
**Figure S22.**	Fragments of HPLC-MS ion chromatograms of CYP3A4 baculosome and control samples supplemented with vomilenine.	**29**
**Figure S23.**	HPLC-UV spectra of vomilenine and putative products of its CYP3A4 oxidation.	**30**

Figure S24. Fragments of HPLC-MS chromatograms of the extract of *N. benthamiana* plants transiently expressing *CYP3A4* or *GFP* (control) and supplemented with vinorine. **31**

S3. References **33**

Supplement

S1. Materials and Methods

S1.1 Genetic material, constructs and chemicals

The sequence of human CYP3A4[S1] employed in the transient expression experiments was optimized using the Mr. Gene GmbH (Regensburg, Germany) algorithm and synthesized by Mr. Gene GmbH. CYP3A4 BACULOSOMES® Plus Reagent was purchased from Thermo Fisher Scientific (USA). Genetic constructs used for transient expression were described previously[S2, S3]. Authentic samples of the investigated compounds: vinorine, perakine, vomilenine, vellosimine, raucaffrinoline and 10-deoxysarpagine were obtained from an in-house collection of indole alkaloids.

S1.2 CYP3A4 baculosome assays

In vitro assays with recombinant CYP3A4 (CYP3A4 BACULOSOMES® Plus Reagent, Thermo Fisher Scientific, USA) were carried out in 250 µl reaction volume containing 10 µl of the baculosome preparation, 0.25 mM vinorine, 2 mM NADPH and 0.1M KPi, pH 7.4. Samples were incubated at 37 ^{o}C in open reaction tubes to facilitate oxygen access. Subsequently, each sample was divided into two sub-samples of 125 µl. The first was supplemented with 10 µl of deionized water and 125 µl of ice-cold acetonitrile (ACN), vortexed and centrifuged at 17,000 × *g* for 20 min before HPLC-ESI-MS analysis. The second sub-sample was supplemented with 7 µl of the $NaBH_4$ solution (1 % in 10 mM NaOH (w/v), freshly prepared) and incubated for 1–3 min. Afterwards, 3 µl of 0.1 N HCl were added. The sample was diluted with 125 µl of ACN and centrifuged at 17,000 × *g* for 20 min before HPLC-ESI-MS analysis.

The relative yield of a single product was determined as a percentage of the substrate (vinorine) content in the control samples (samples with enzymes inactivated by boiling, which were not

reduced with sodium borohydride), calculated on the base of areas of corresponding ionized product peaks on HPLC-MS chromatogram. All assays were performed in triplicate.

S1.3 Preparative extraction of vinorine 19*R*, 20*R*-epoxide

Individual baculosome samples, prepared as described above and incubated for 2, 4 and 8 h (3 × 40 samples, respectively), were combined and extracted twice with the equal volume of ethyl acetate. The organic extracts were combined and filtered through an anhydrous sodium sulfite layer; the solvent was evaporated under vacuum at 40 ^{o}C. The extracts were dissolved in the mixture of MeOH and CH_2Cl_2 (1:1) and loaded onto the 0.2 mm silica gel ALUGRAM1 Xtra Sil G/UV 254 TLC plate, 20 × 20 cm (Macherey-Nagel, Germany). The extracted compounds were resolved in the $CHCl_3$/n-hexane/diethylamine (6:3:1) solvent system and detected by their absorption at 254 nm and by colour reaction after spraying with the ceric ammonium sulfate reagent. Compounds characterized by the R_f value of 0.37 (vellosimine), 0.51 (vinorine 19*R*, 20*R*-epoxide) and 0.61 (vinorine) were eluted from the silica gel, dried and resolved again in the $CHCl_3$/MeOH/ammonia solution (25%) (8:2:0.1) solvent system yielding 0.16 mg of pure vinorine 19*R*, 20*R*-epoxide (R_f 0.73). Additionally, vinorine (R_f 0.76; 0.36 mg;) and vellosimine (R_f 0.62; 1,34 mg) were isolated. An amount of the substances was calculated using the linear part of the calibration curve built for the standard vinorine solutions at $OD_{270.}$

Vinorine 19*R*, 20*R*-epoxide: whitish substance; 1H and 13C NMR, Table 1; UV (44 % (v/v) acetonitrile in 0.1 % (v/v) formic acid, concentration of the substance was calculated using the linear part of the calibration curve built for the standard vinorine solutions at OD_{270}) λ_{max} (log ε): 219 (4.47); 260 (3.83). HPLC-ESI-MS *m/z* (rel. int., %) 351 [M+H$^+$] (100); HPLC-ESI-HR-MS *m/z* calcd. for $C_{21}H_{23}N_2O_3$+H$^+$: 351.17032 [M+H$^+$]; found 351.17044.

S1.4 Transient expression of *CYP3A4* in planta

Transient expression was carried out as described previously[S2, S3]. In brief, liquid cultures of *A. tumefaciens* (strain GV3101) harbouring the CYP3A4 expression cassette and the GFP::licBM3 reporter gene (control culture) were infiltrated into the 1st and 2nd fully developed upper leaves of *N. benthamiana*. Vinorine was prepared as 8 mg/ml (23.9 mM) stock solution in MeOH and added to the bacterial suspension, to the final concentration of 0.2 mg/ml (0.6 mM), immediately before injection. To provide a positive control of CYP3A4 in planta activity, loratadine was infiltrated as described previously[S2, S3]. The plants were incubated under greenhouse conditions. Administration of the CYP3A4 substrates was repeated on the 5th dpi and the biomass was harvested on the 8th dpi.

For HPLC-ESI-MS analysis, explants of 25 mm in diameter were excised from the centre of the infiltration area and freeze-dried in the Eppendorf tubes. Immediately after, the samples were ground and defatted with 1 ml of petroleum ether for 5 min under sonication. Following centrifugation for 5 min at 17,000 × *g*, the supernatants were removed and the residue was dried in a fume hood for 15–20 min. The residue was then extracted with 500 µl of MeOH for 5 min under sonication and for further 30 min on a shaker (750 rpm) at 40 °C. After centrifugation, the supernatants were collected and the residue was re-extracted with 250 µl of MeOH for 10 min at 750 rpm and 40 °C. The methanol extracts were combined and the solvent evaporated using a centrifugal vacuum concentrator. The residues were taken up into 500 µl of 0.1 N HCL and the water phase was extracted with 500 µl of ethyl acetate for 10 min at 1,250 rpm and 25 °C. After centrifugation, the organic fraction was discarded and the pH of the water phase was adjusted to alkaline by adding 10 µl of 33 % ammonia solution. The water phase was extracted twice with 500 µl of ethyl acetate for 10 min at 1,250 rpm and 25 °C. The organic

fractions were combined and evaporated. The residues were dissolved in methanol and analysed by HPLC-ESI-MS.

The relative yield of alkaloids was determined as a percentage of the total amount of alkaloids in the sample, calculated on the base of areas of the corresponding ionized product peaks on HPLC-MS chromatogram or OD_{278} absorbance on HPLC chromatogram. All assays were performed minimum in triplicate.

S1.5 Analytical assays

HPLC-MS analyses were carried out with the 1260 Infinity HPLC system (G4226A micro vacuum degasser, G1312B binary pump, G1329B autosampler, G1316C column thermostat, G4212B diode array detector) coupled to the G6120B quadrupole mass spectrometry detector (Agilent). For alkaloid separation, we used the analytical column Poroshell 120 SB-C18 (Agilent) 3×150 mm, 2.7 μm and the mobile phase consisting of 0.1 % (v/v) formic acid (A) and acetonitrile (B). For the separation, the following binary gradient elution programme was applied (% B): 10 within 2 min; 10–100 within 18 min. The column was flushed with 100 % B for 8 min and re-equilibrated with 15 % B for 17 min. The flow rate was set to 0.5 ml/min and detection conducted at 245 and 278 nm. Metabolites were detected in the positive API-ES mode with the following parameters: positive ionization mode with a full scan MS measurement (m/z, 100–800); desolvation gas, nitrogen at 350 °C and 720 l/h; nebulizer pressure, 35 psi; capillary voltage, 3,000 V.

^{1}H and ^{13}C NMR, ^{13}C-DEPT, CLIP-COSY, NOESY, ^{13}C-HSQC and ^{13}C-HMBC spectra of vinorine 19*R*, 20*R*-epoxide, vinorine and vellosimine were recorded using the AVANCE III HD instrument (Bruker, USA) equipped with the QCI CryoProbe at 700 MHz (^{1}H) and 176 MHz (^{13}C).

S1.6 Computational design

The X-ray structure of the human microsomal cytochrome P450 3A4 (pdb-code, 1TQN[S4]) was used for docking studies of vinorine. All calculations were performed with MOE 2019.01.01 (https://www.chemcomp.com/). Hydrogen atoms were added to the X-ray structure with the help of the protonate-3d tool. Hydrogen peroxide was added manually to the heme iron ion. Vinorine was constructed and energy optimized with the AMBER14-EHT force field (a special modified AMBER force field embedded in MOE) and the Born solvation equation.

Figures

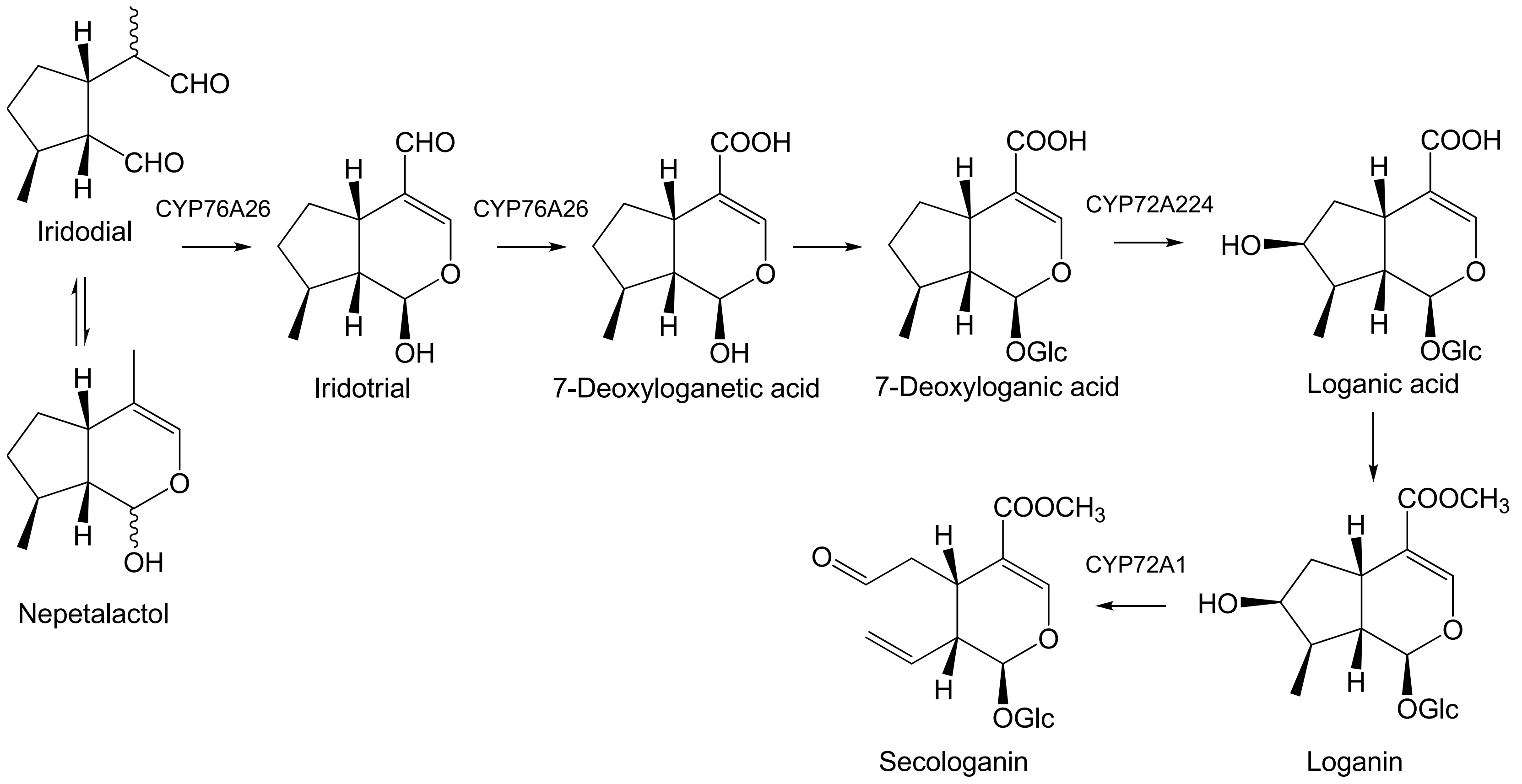

Figure S1. Selected routes of iridoid biosynthesis pathway in *Catharanthus roseus* from iridodial to secologanin [S5,S6] exhibiting the diversity of CYP-mediated reactions (reactions performed by CYPs are marked).

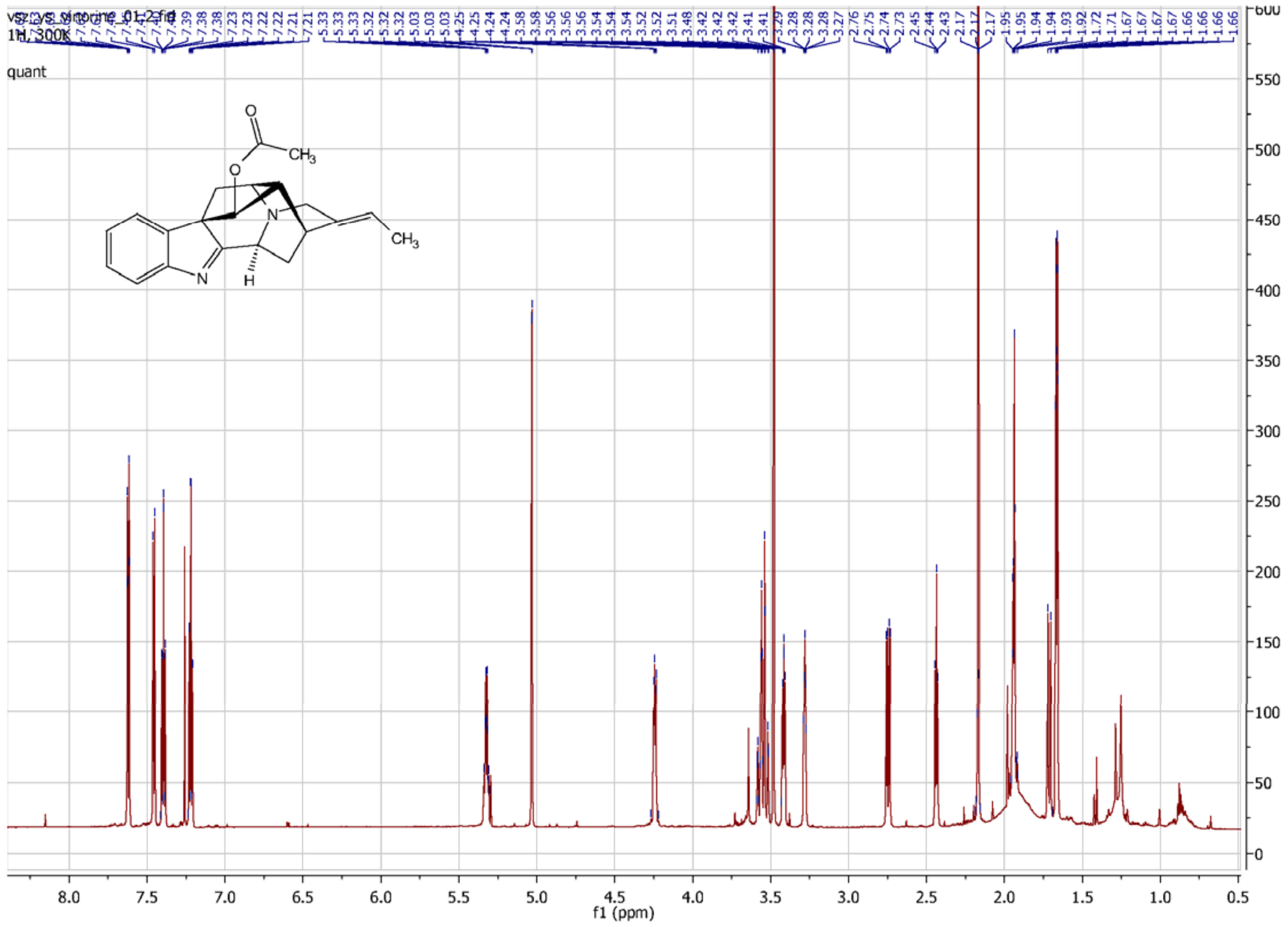

Figure S2. ^{1}H NMR spectrum of vinorine in $CDCl_3$.

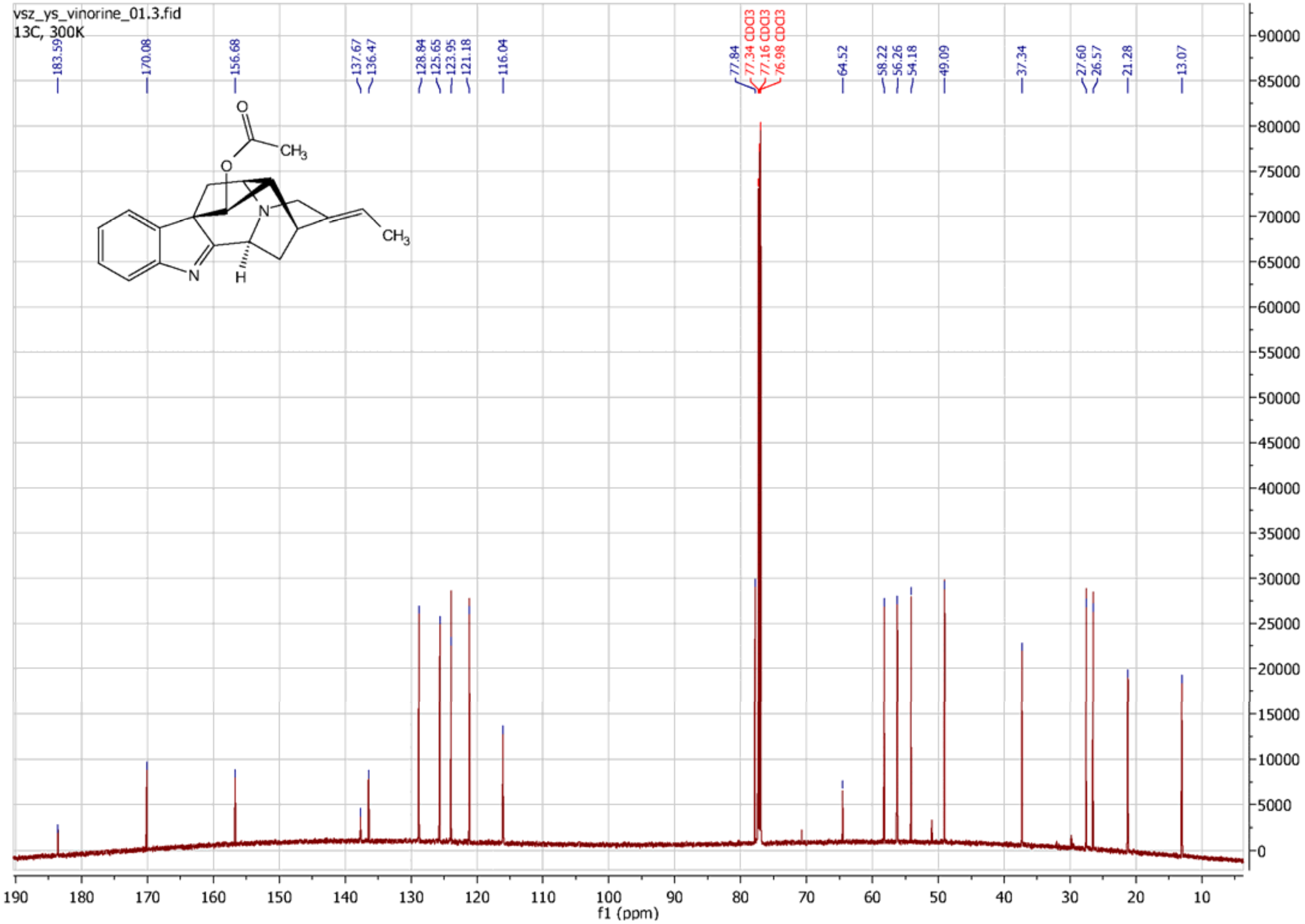

Figure S3. ^{13}C NMR spectrum of vinorine in $CDCl_3$.

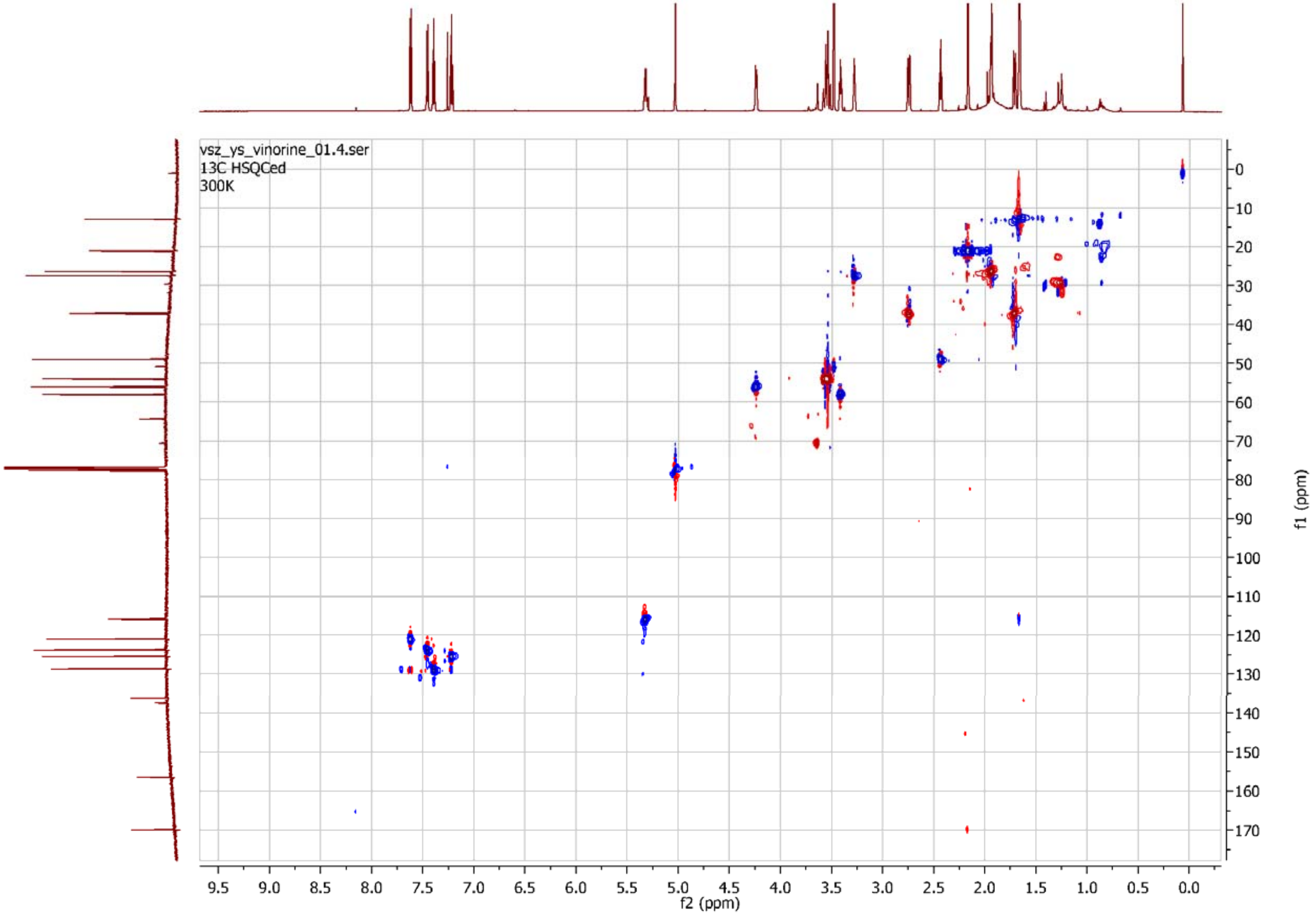

Figure S4. ^{1}H–^{13}C HSQC spectrum of vinorine in $CDCl_3$.

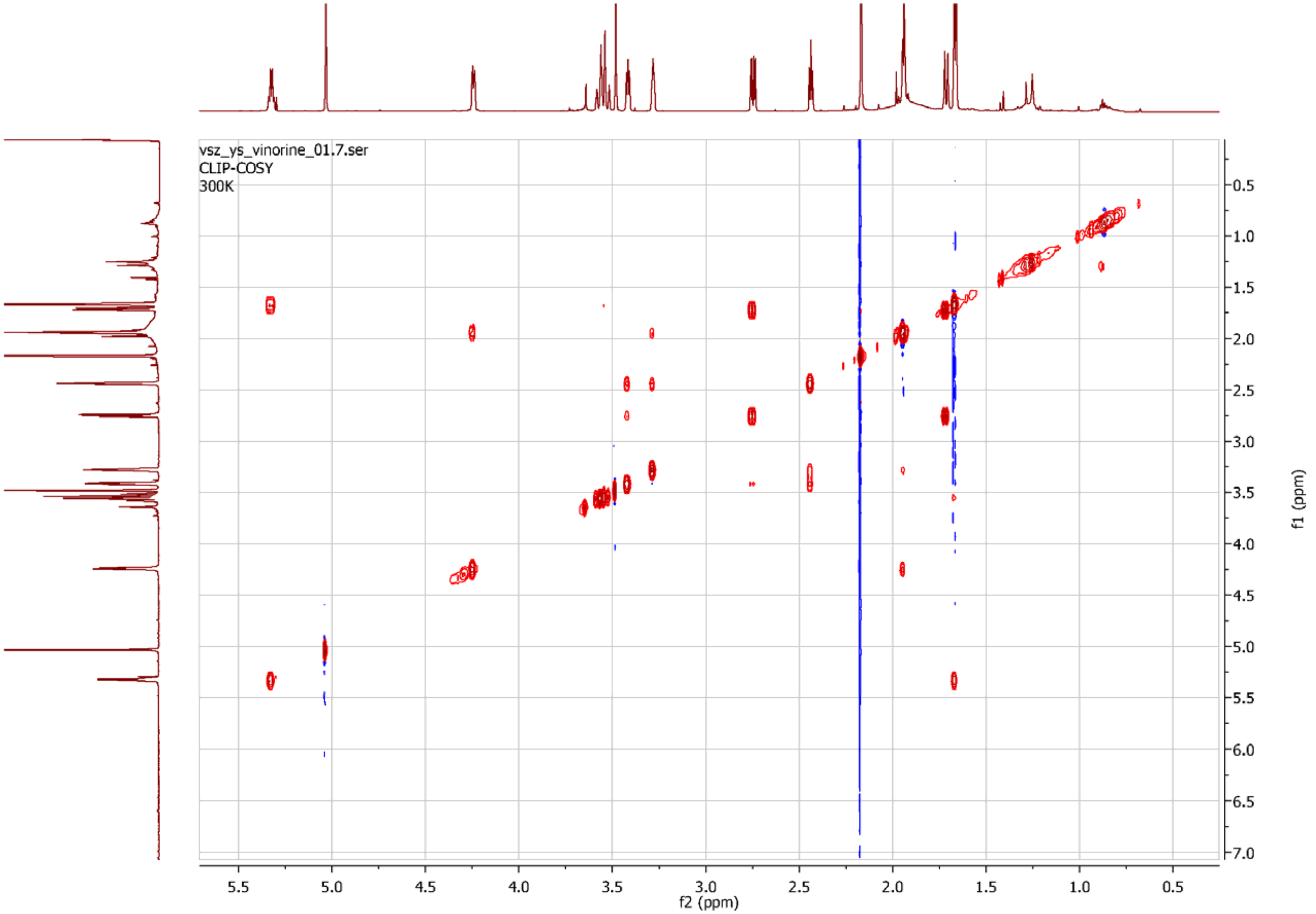

Figure S5. CLIP-COSY spectrum of vinorine in $CDCl_3$.

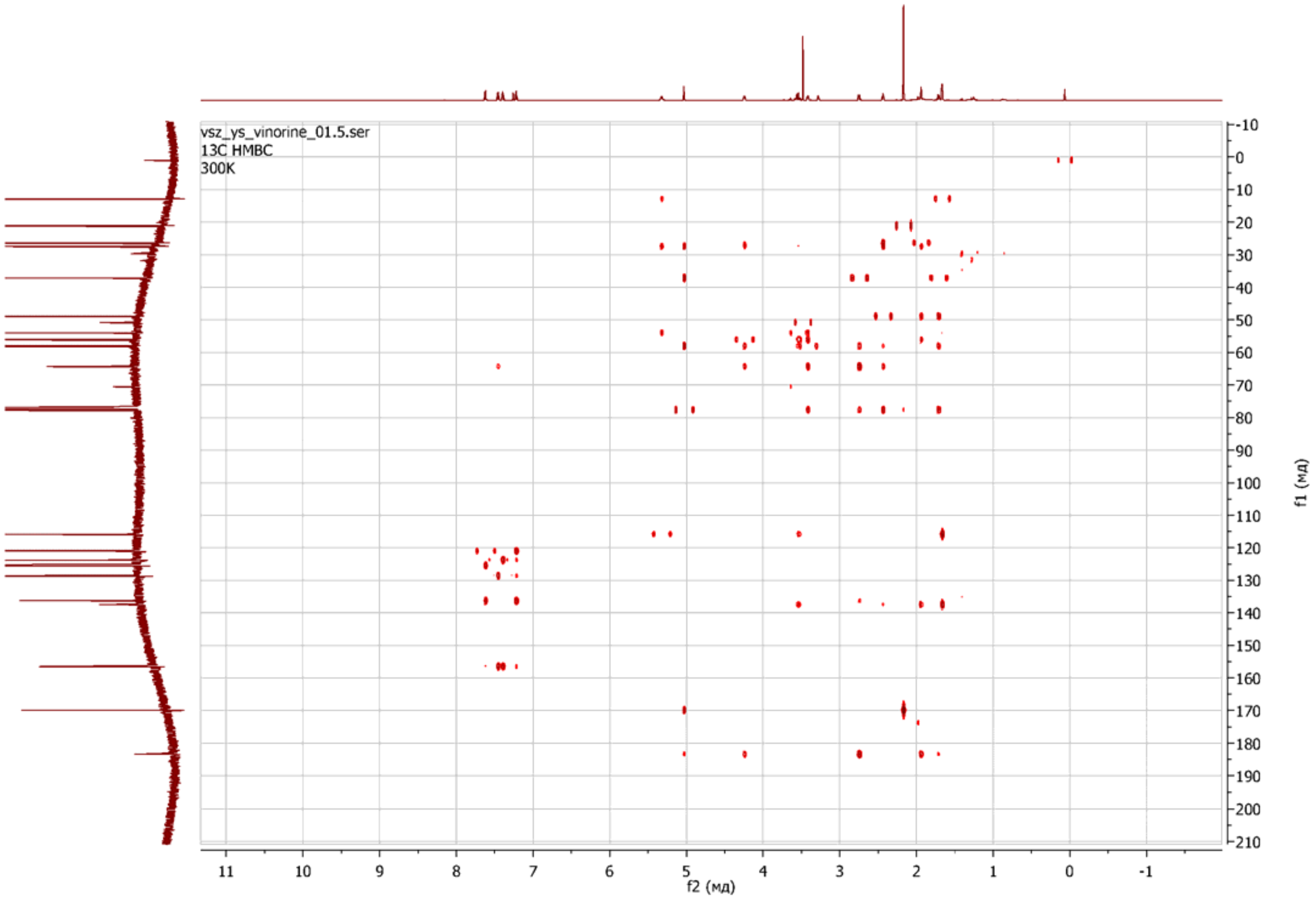

Figure S6. ^{1}H–^{13}C HMBC spectrum of vinorine in $CDCl_3$.

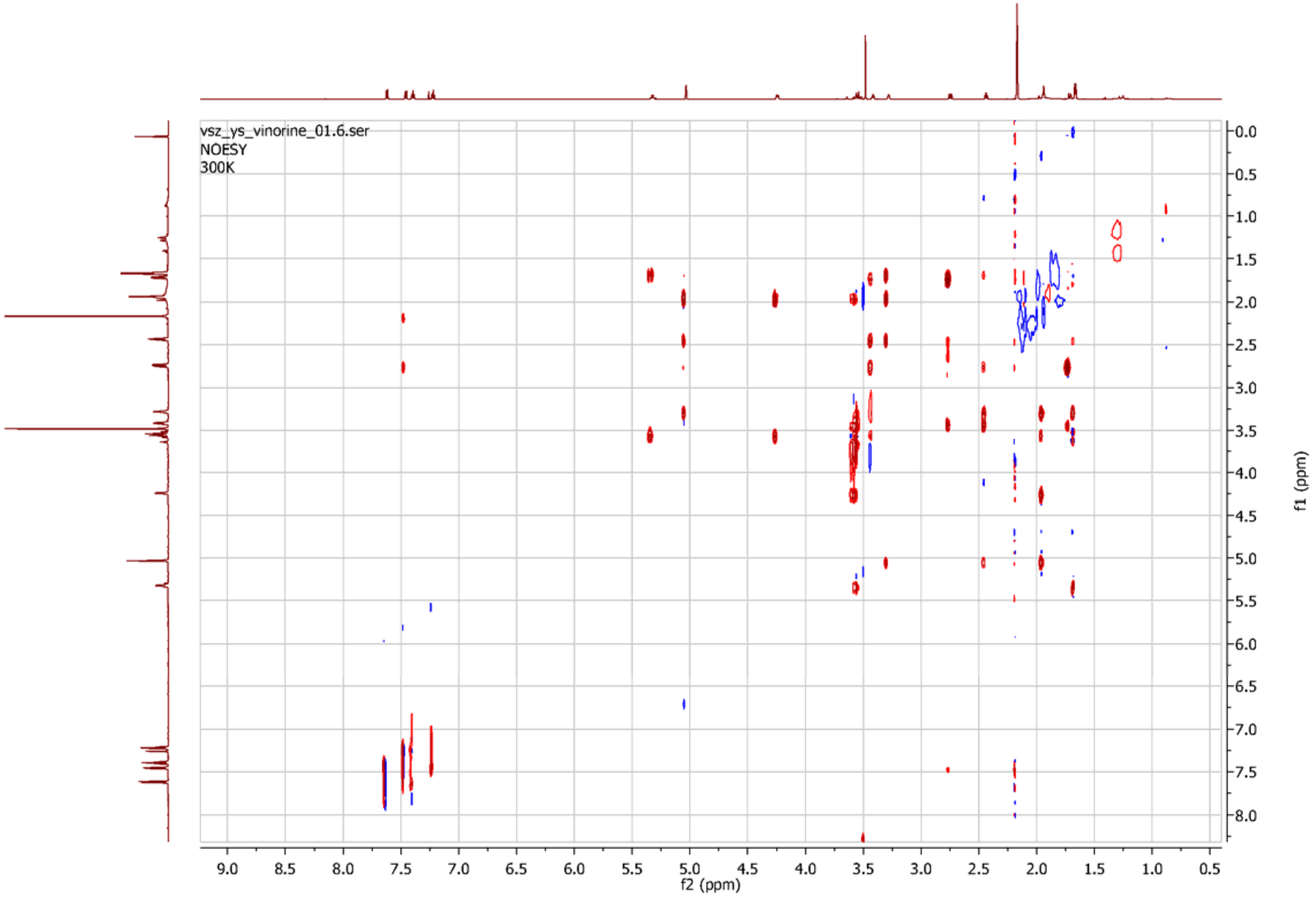

Figure S7. NOESY spectrum of vinorine in $CDCl_3$.

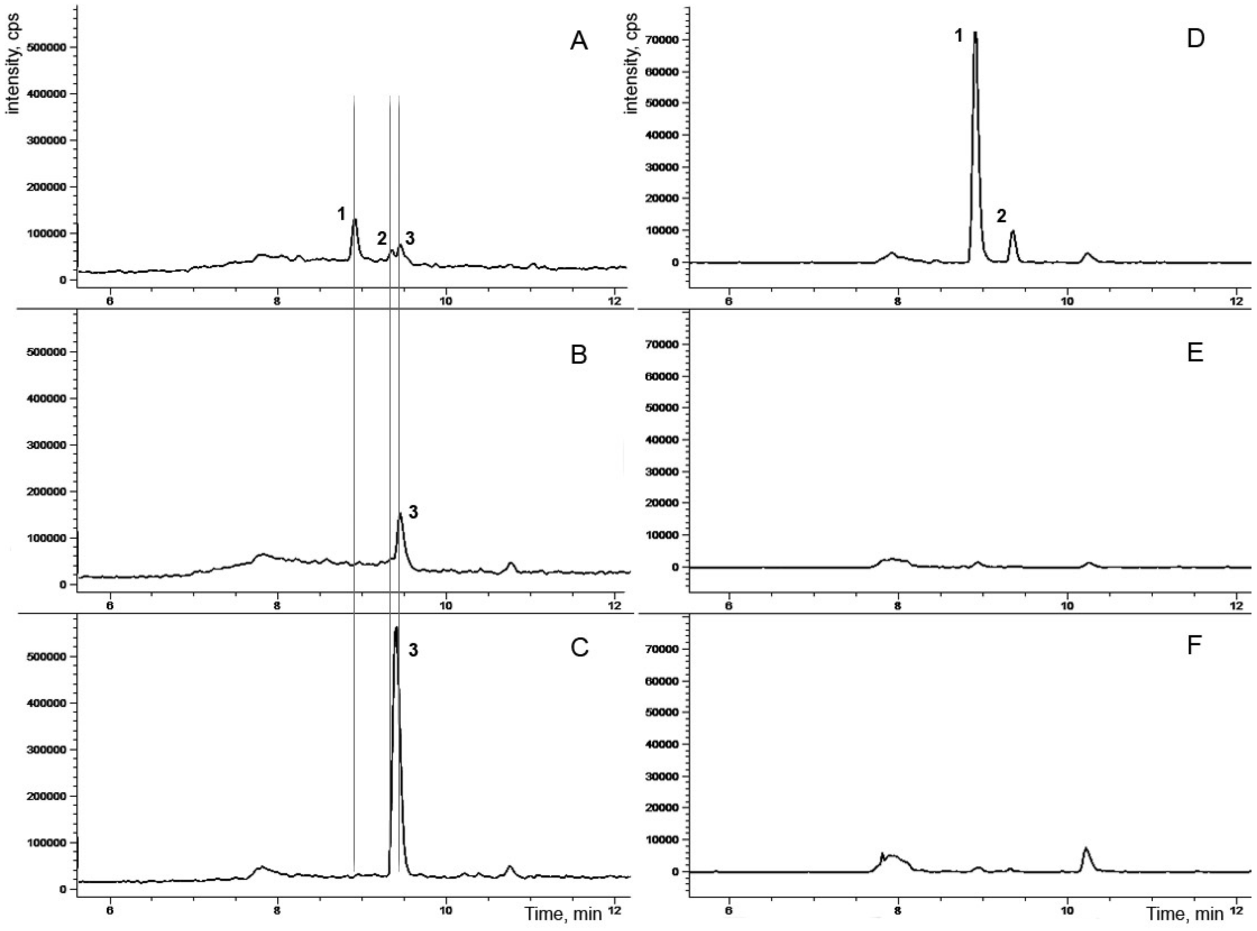

Figure S8. Fragments of HPLC-MS ion chromatograms of CYP3A4 baculosome samples. A) CYP3A4 baculosome preparation supplemented with vinorine and NADPH; B) CYP3A4 baculosome preparation supplemented with vinorine and lacking NADPH (control 1); C) CYP3A4 baculosome preparation inactivated by boiling and supplemented with vinorine and NADPH (control 2); D-F) respective selective extraction of m/z 351 ion chromatogram. 1, Vinorine 19*R*, 20*R*-epoxide (m/z $[M+H]^+$ 351); 2, vomilenine (m/z $[M+H]^+$ 351); 3, vinorine (m/z $[M+H]^+$ 335).

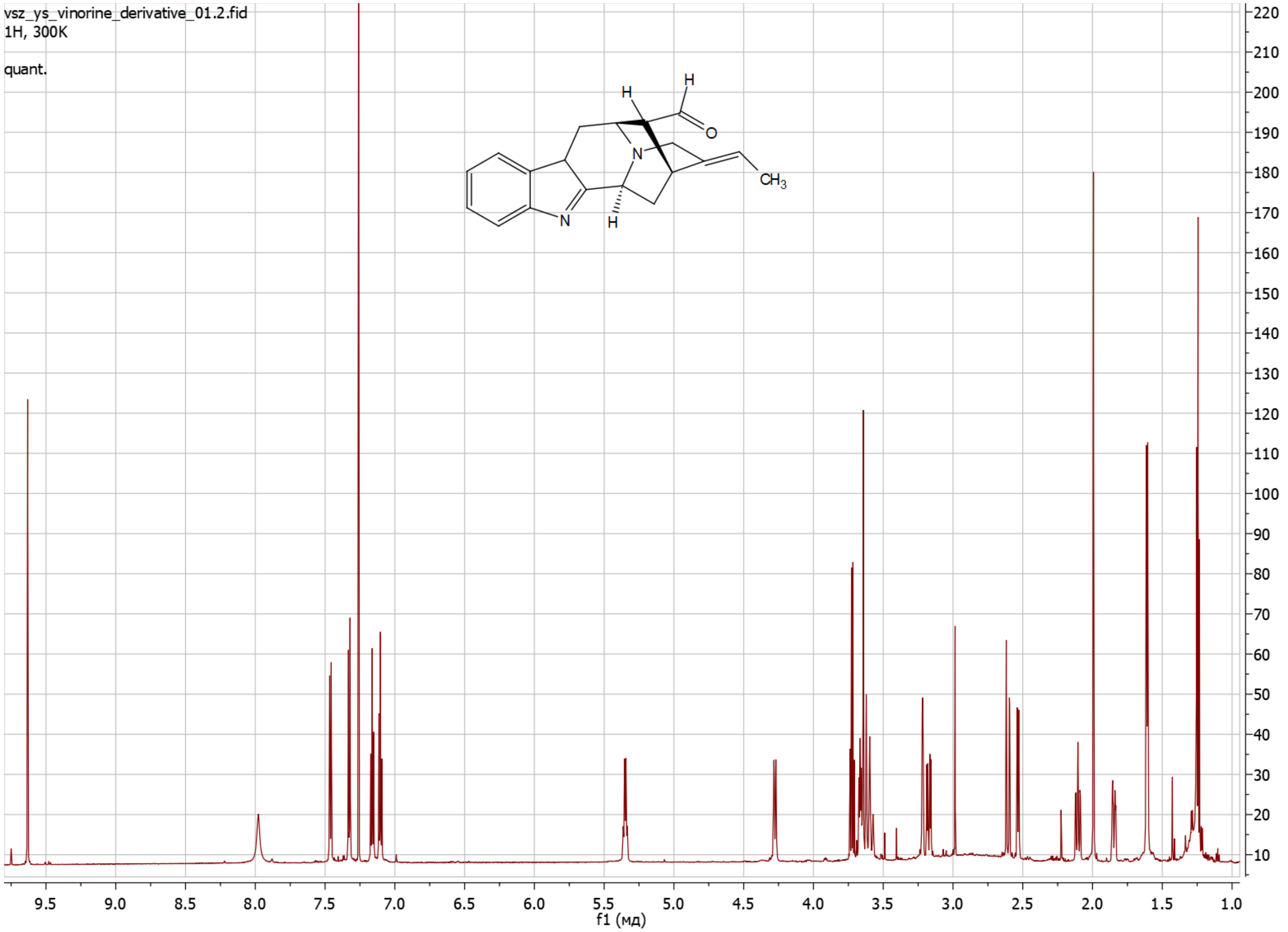

Figure S9. ^{1}H NMR spectrum of vellosimine in $CDCl_3$.

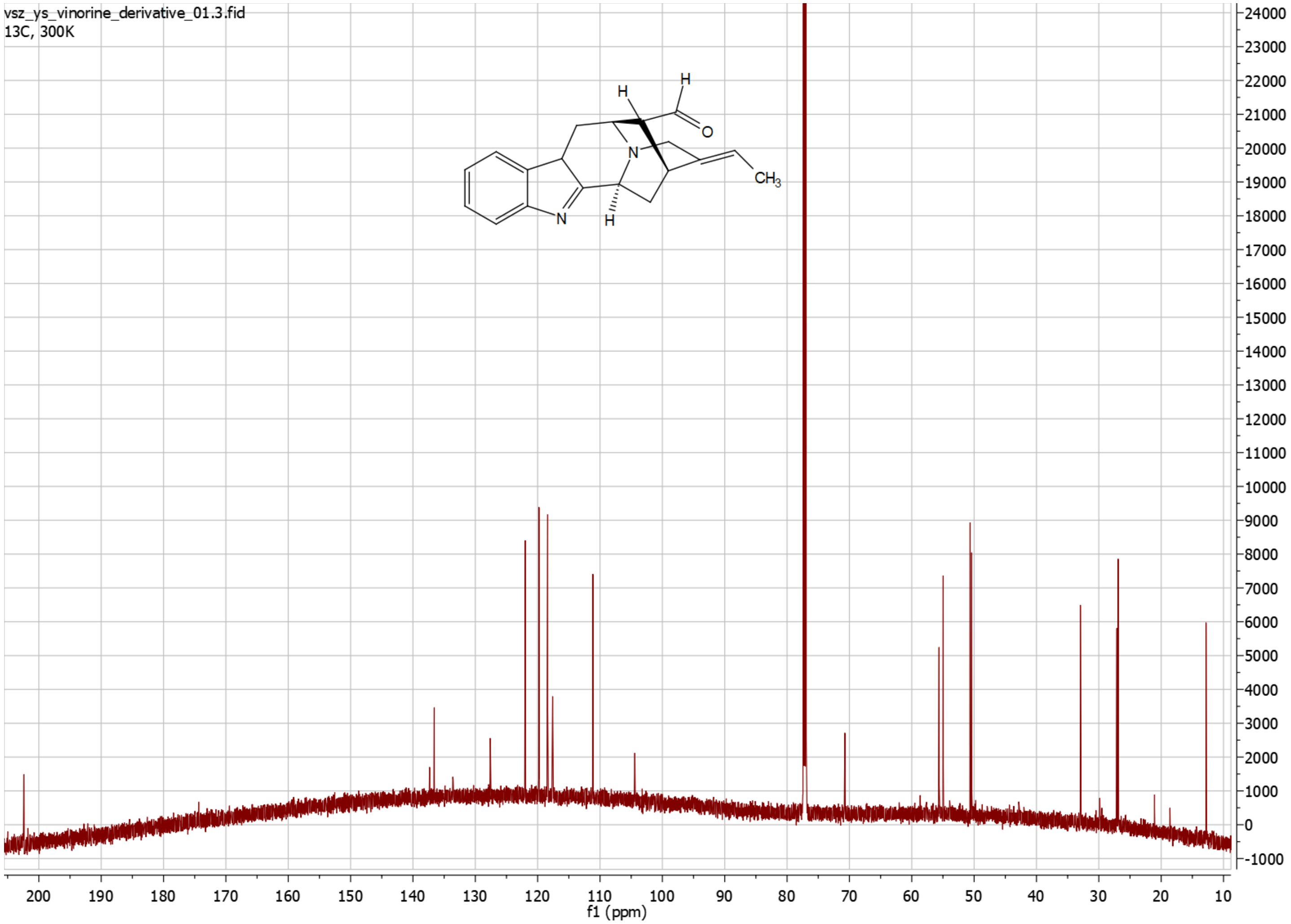

Figure S10. ^{13}C NMR spectrum of vellosimine in $CDCl_3$.

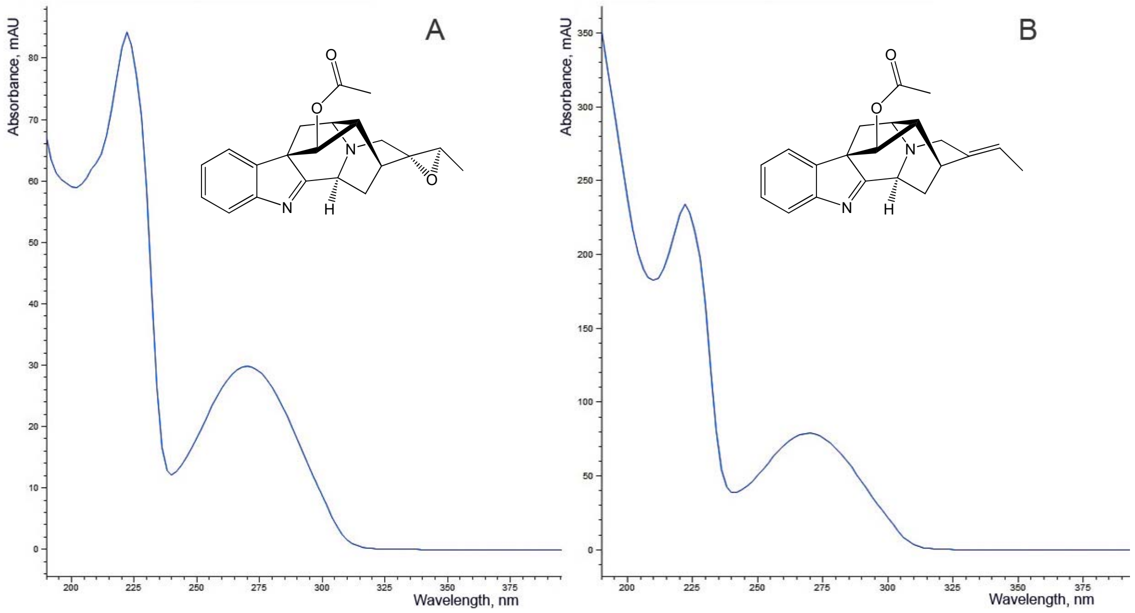

Figure S11. HPLC-UV spectra of vinorine 19*R*, 20*R*-epoxide (A) and vinorine (B).

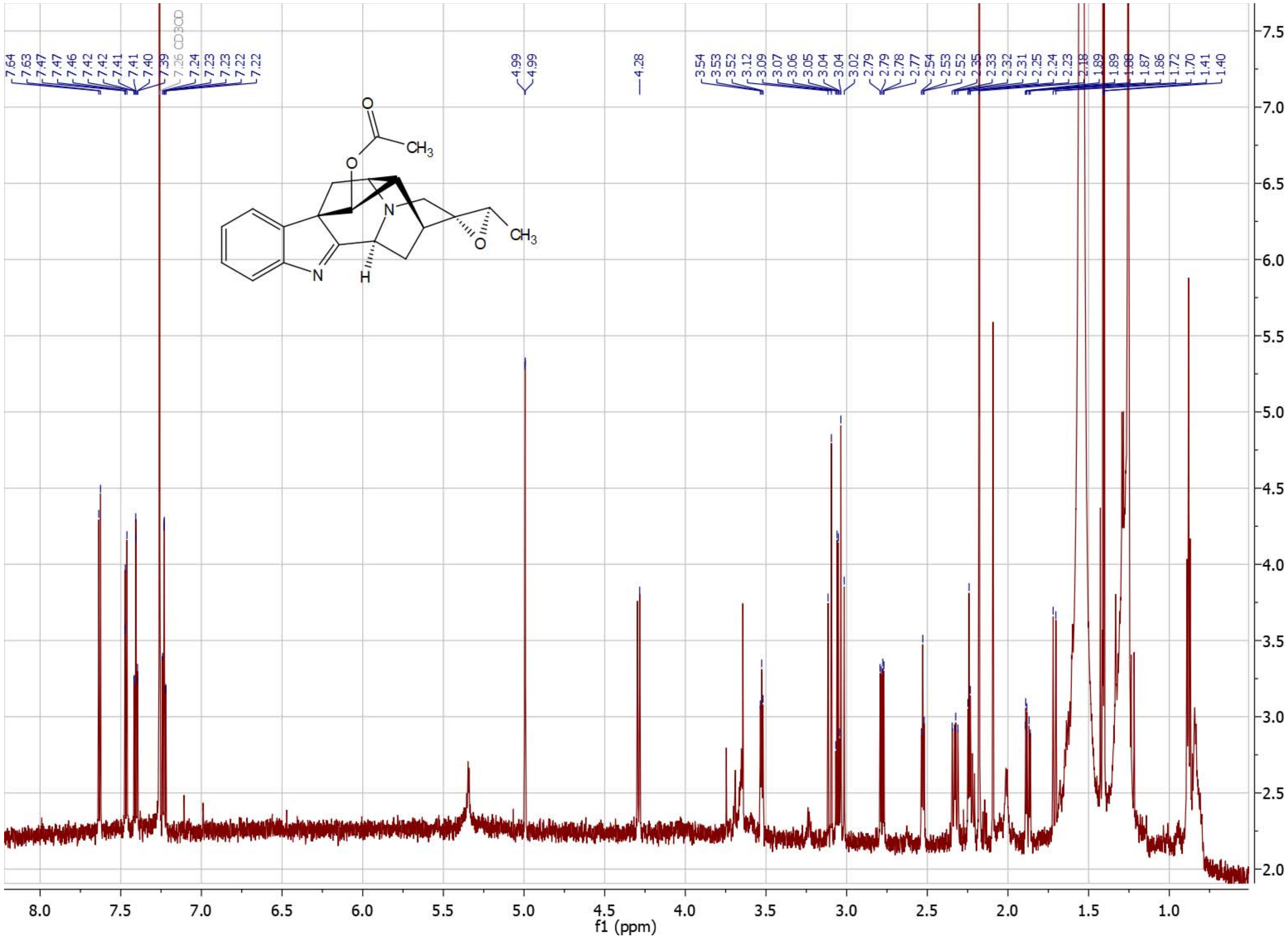

Figure S12. ^{1}H NMR spectrum of vinorine 19*R*, 20*R*-epoxide in $CDCl_3$.

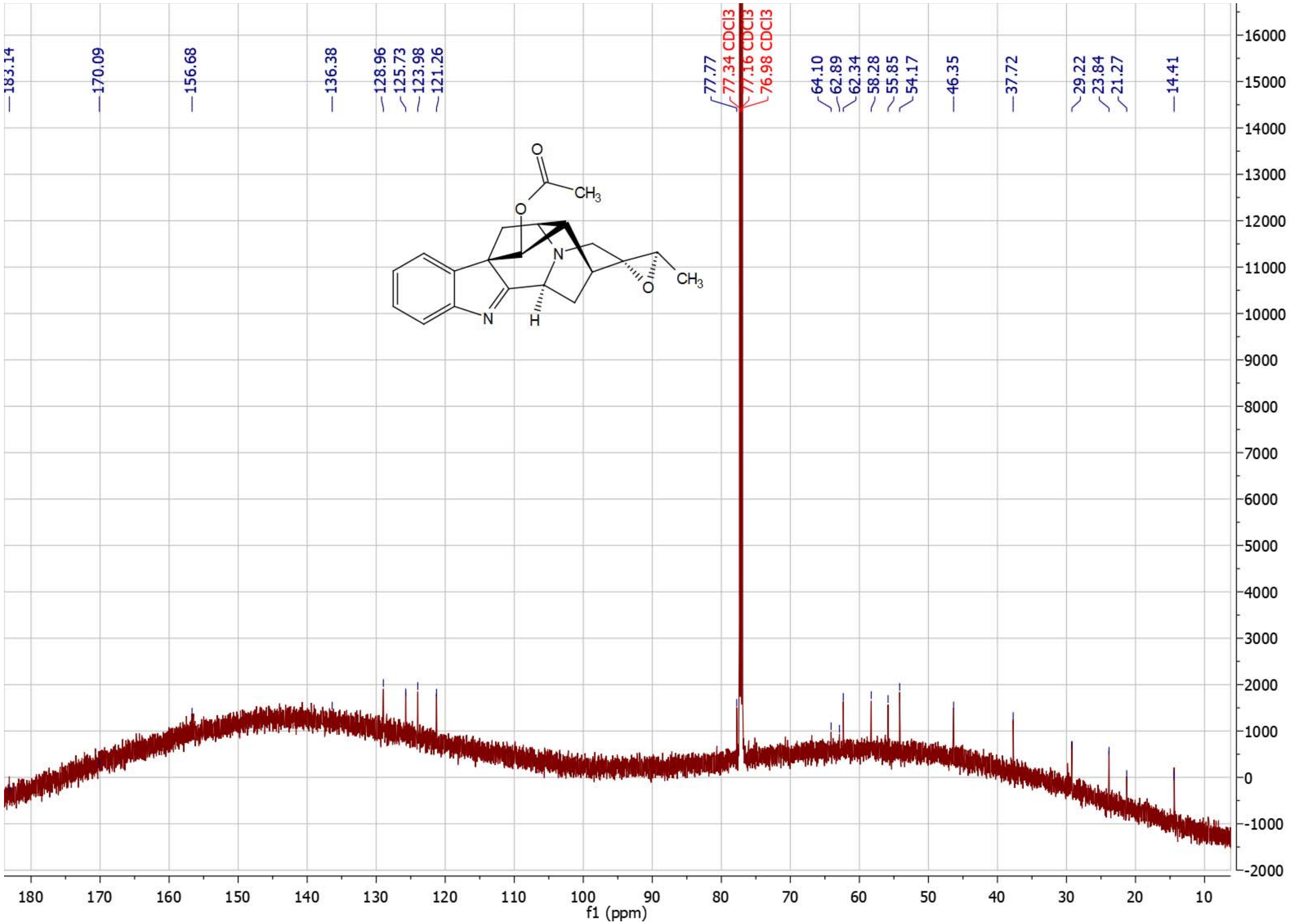

Figure S13. ^{13}C NMR spectrum of vinorine 19*R*, 20*R*-epoxide in $CDCl_3$.

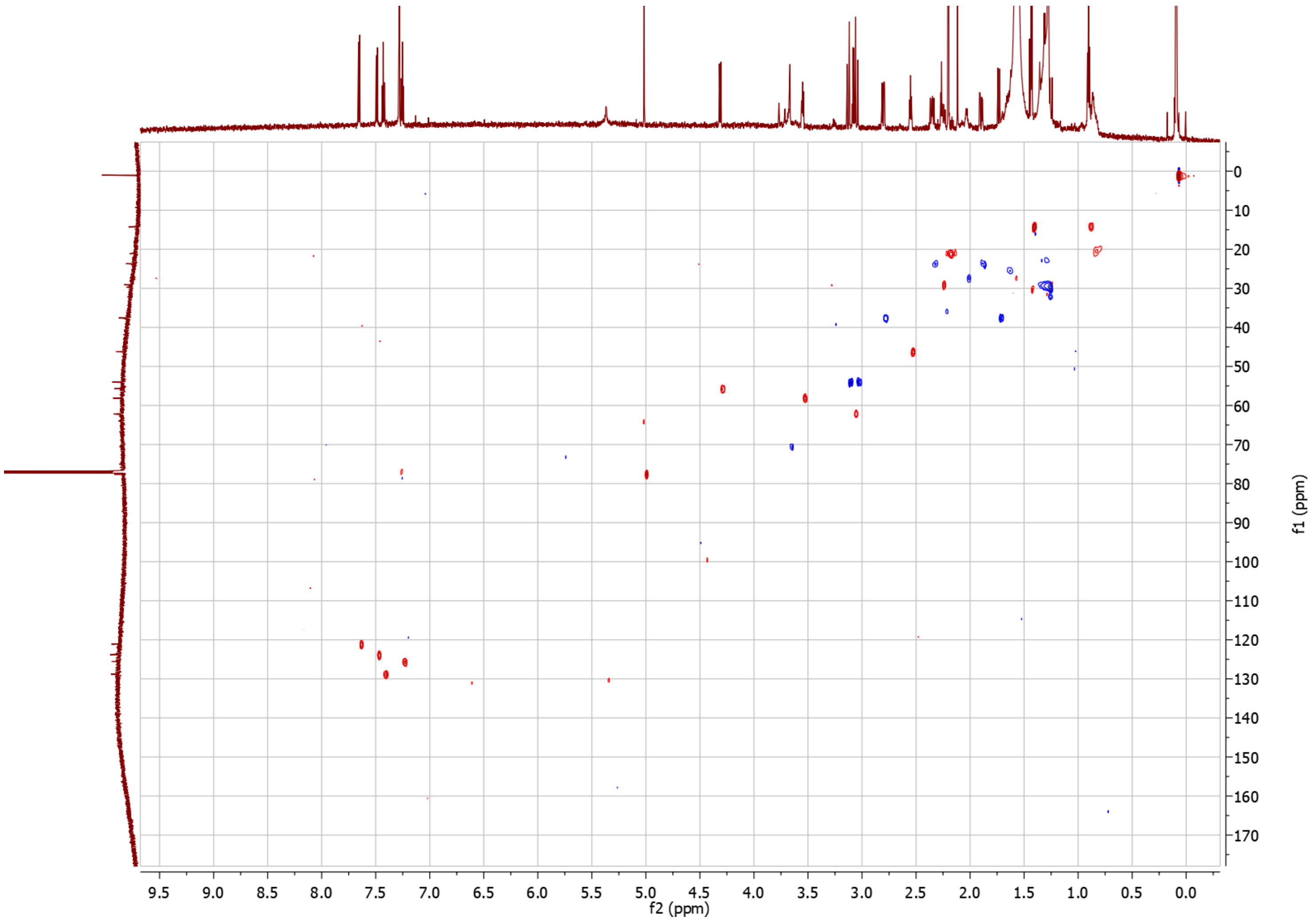

Figure S14. ^{1}H–^{13}C HSQC spectrum of vinorine 19*R*, 20*R*-epoxide in $CDCl_3$.

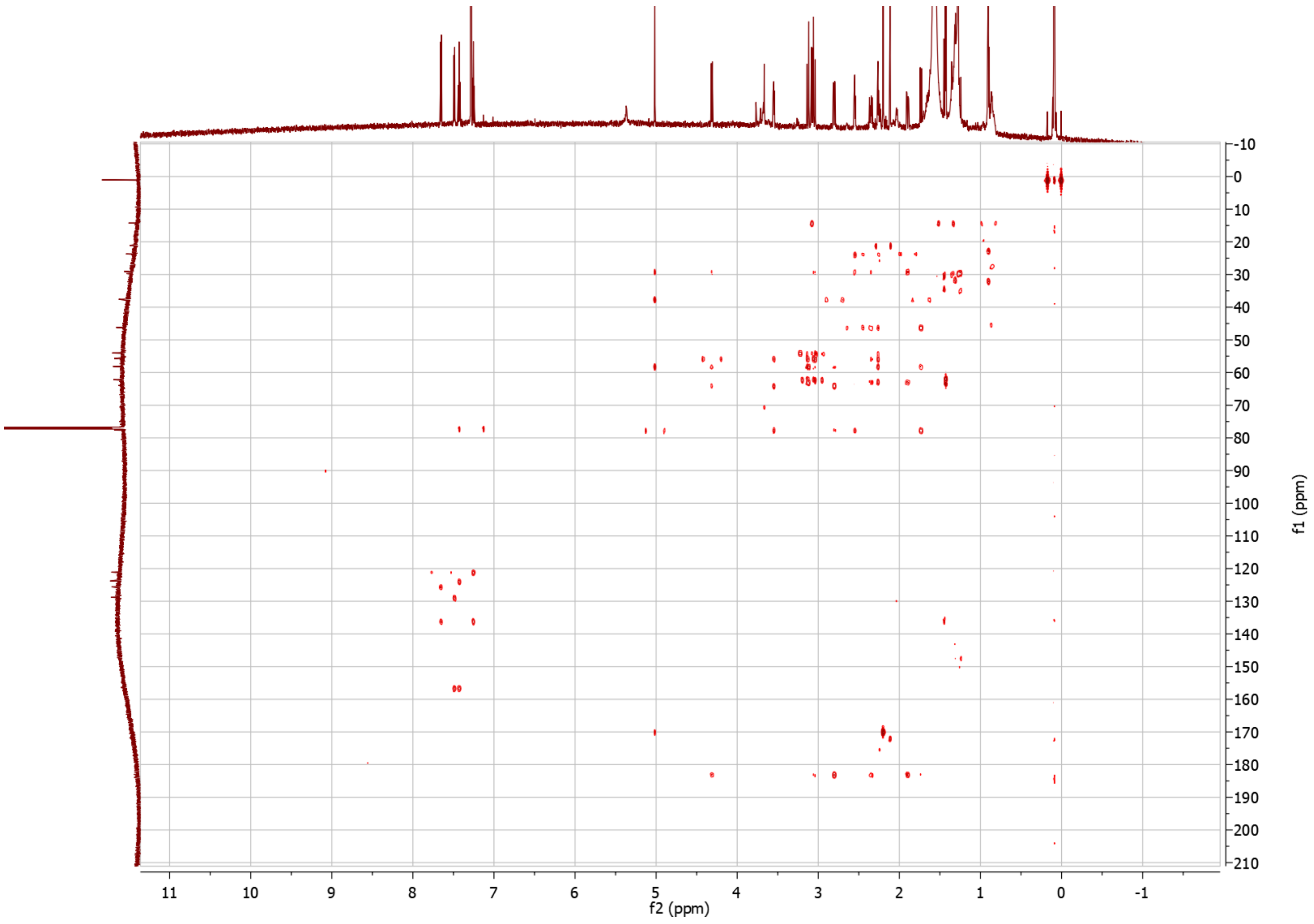

Figure S15. ^{1}H-^{13}C SMBC spectrum of vinorine 19*R*, 20*R*-epoxide in $CDCl_3$.

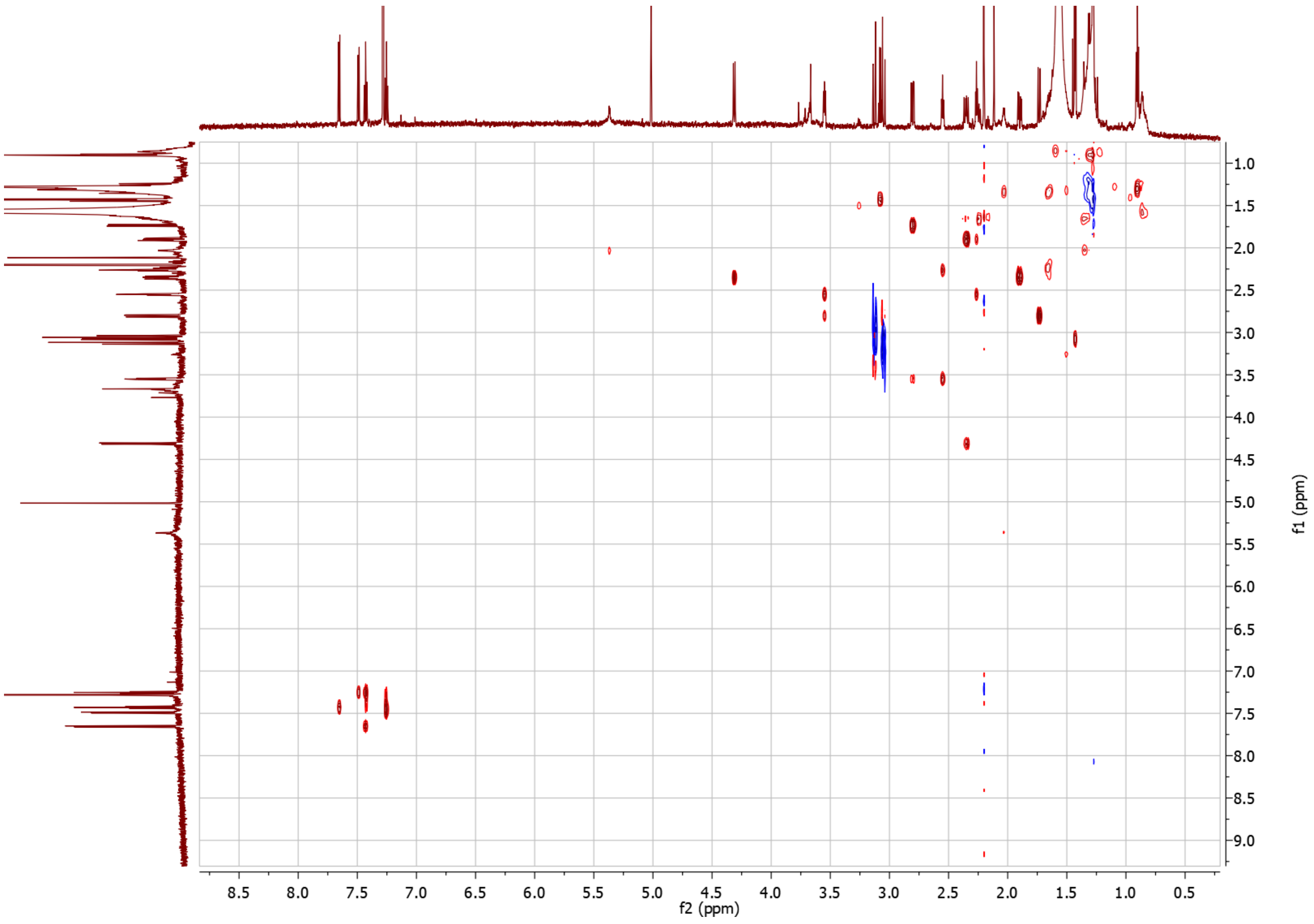

Figure S16. CLIP-COSY spectrum of vinorine 19*R*, 20*R*-epoxide in $CDCl_3$.

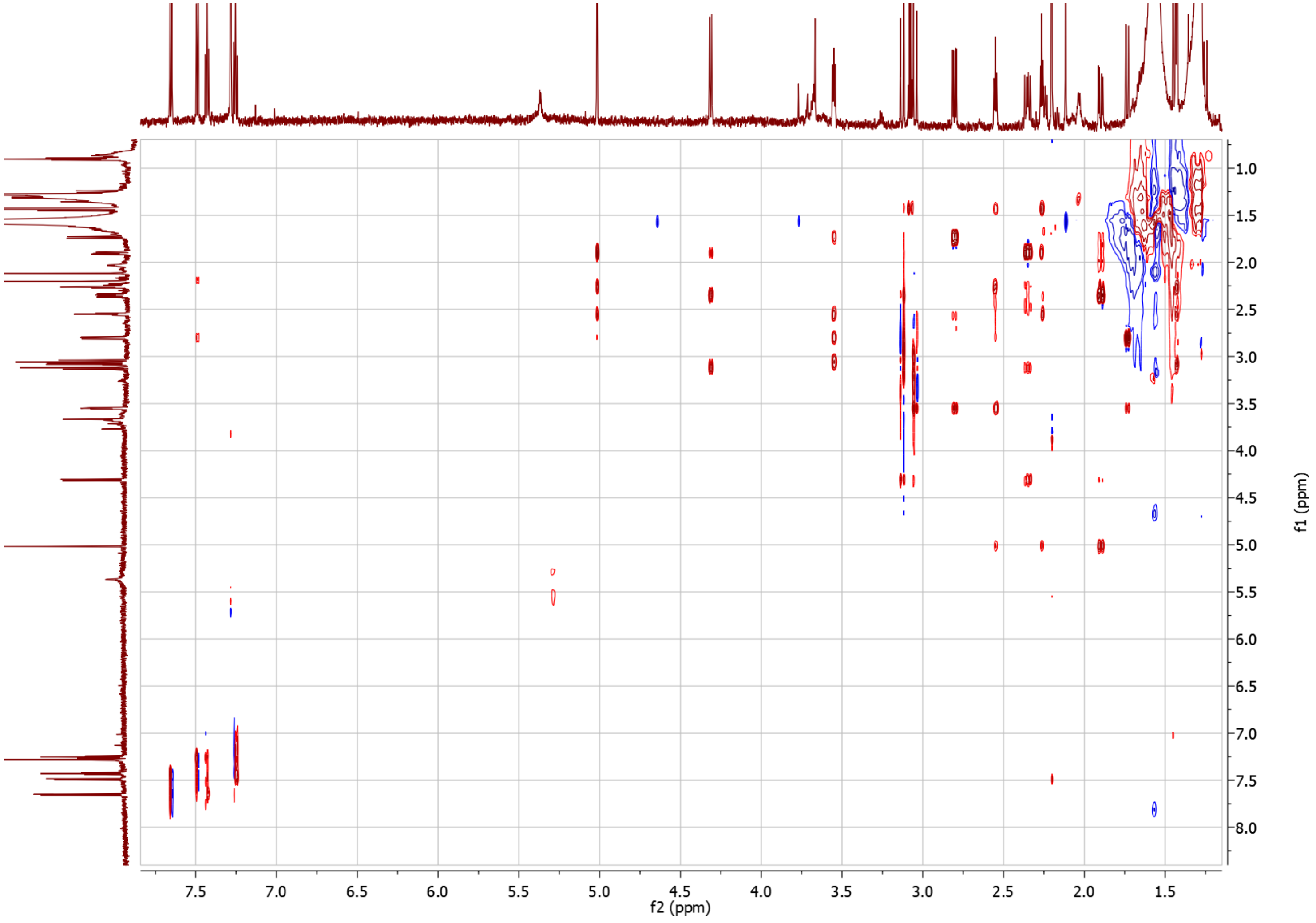

Figure S17. NOESY spectrum of vinorine 19*R*, 20*R*-epoxide in $CDCl_3$.

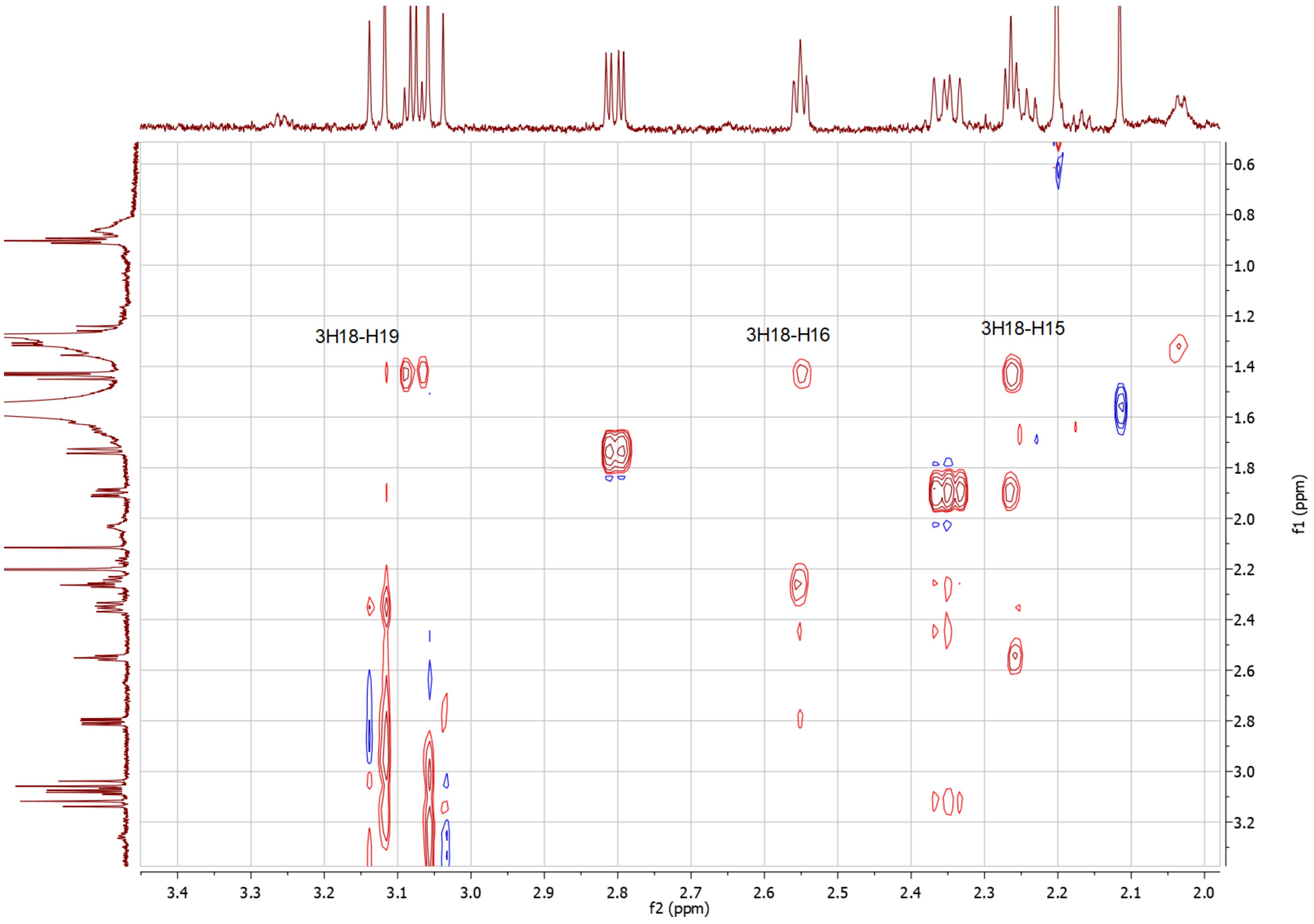

Figure S18. Fragment of NOESY spectrum of vinorine 19*R*, 20*R*-epoxide in $CDCl_3$. The selected proton-proton interactions are marked.

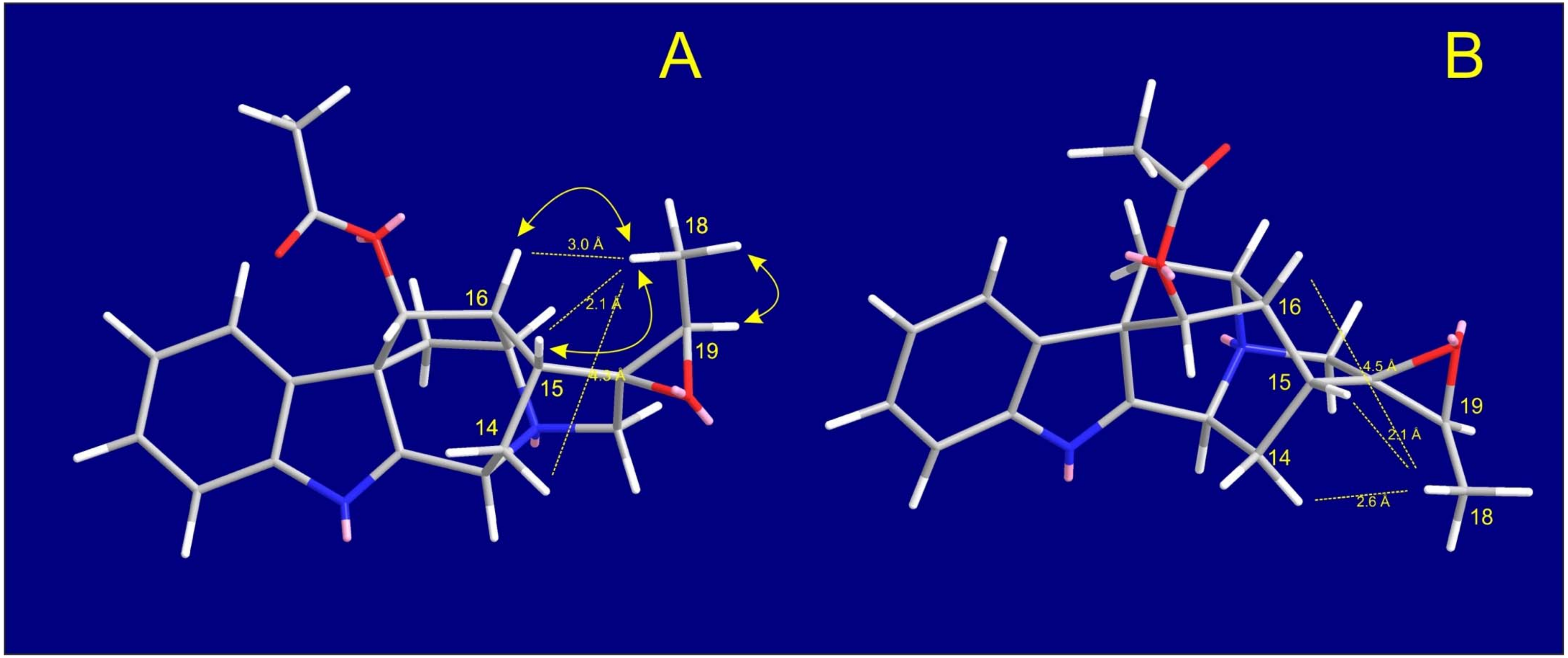

Figure S19. 3D models of vinorine 19, 20-epoxide isomers; selected NOE-interactions are marked with arrows and selected interproton distances are marked with dotted lines. A) vinorine 19*R*, 20*R*-epoxide; B) vinorine 19*S*, 20*S*-epoxide.

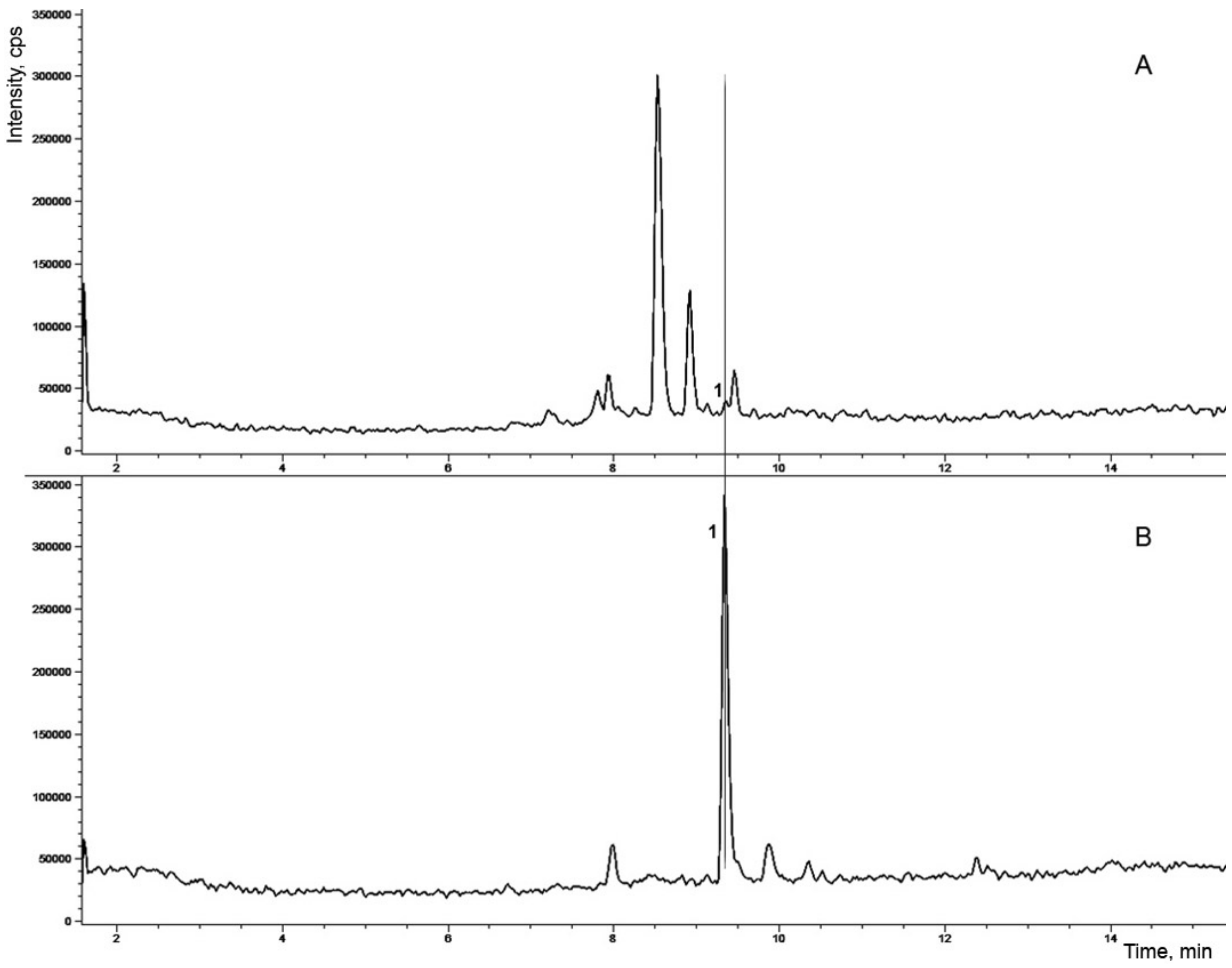

Figure S20. Fragments of HPLC-MS ion chromatograms of CYP3A4 baculosome samples after sodium bvorohydride reduction. A) CYP3A4 baculosome preparation supplemented with vinorine and NADPH; B) CYP3A4 baculosome preparation inactivated by boiling and supplemented with vomilenine and NADPH. 1, Vomilenine (m/z $[M+H]^+$ 351).

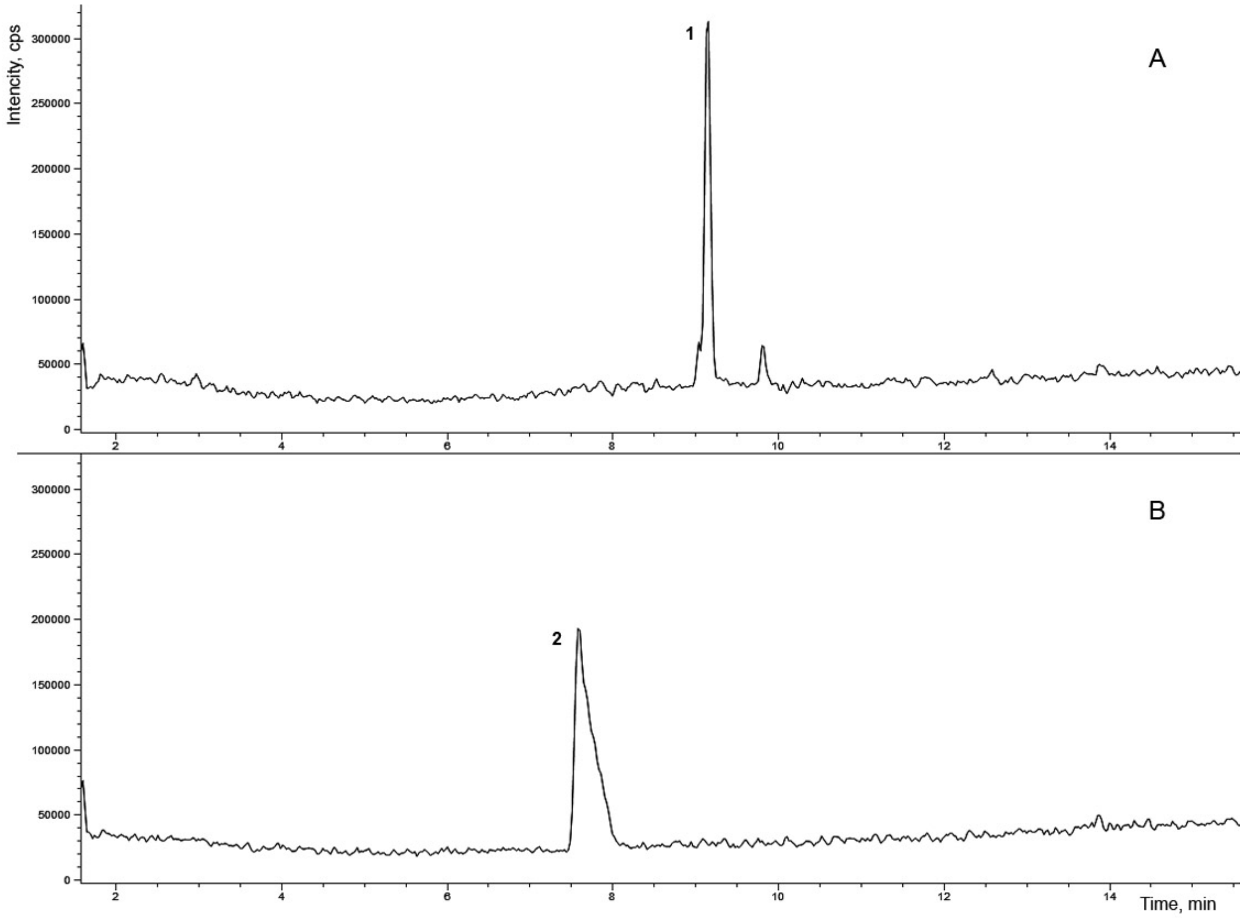

Figure S21. Fragments of HPLC-MS ion chromatograms of CYP3A4 baculosome samples. A) CYP3A4 baculosome preparation supplemented with vomilenine and NADPH; B) CYP3A4 baculosome preparation inactivated by boiling and supplemented with perakine and NADPH. 1, Vomilenine (m/z $[M+H]^+$ 351); 2, perakine (m/z $[M+H]^+$ 369, hydrate).

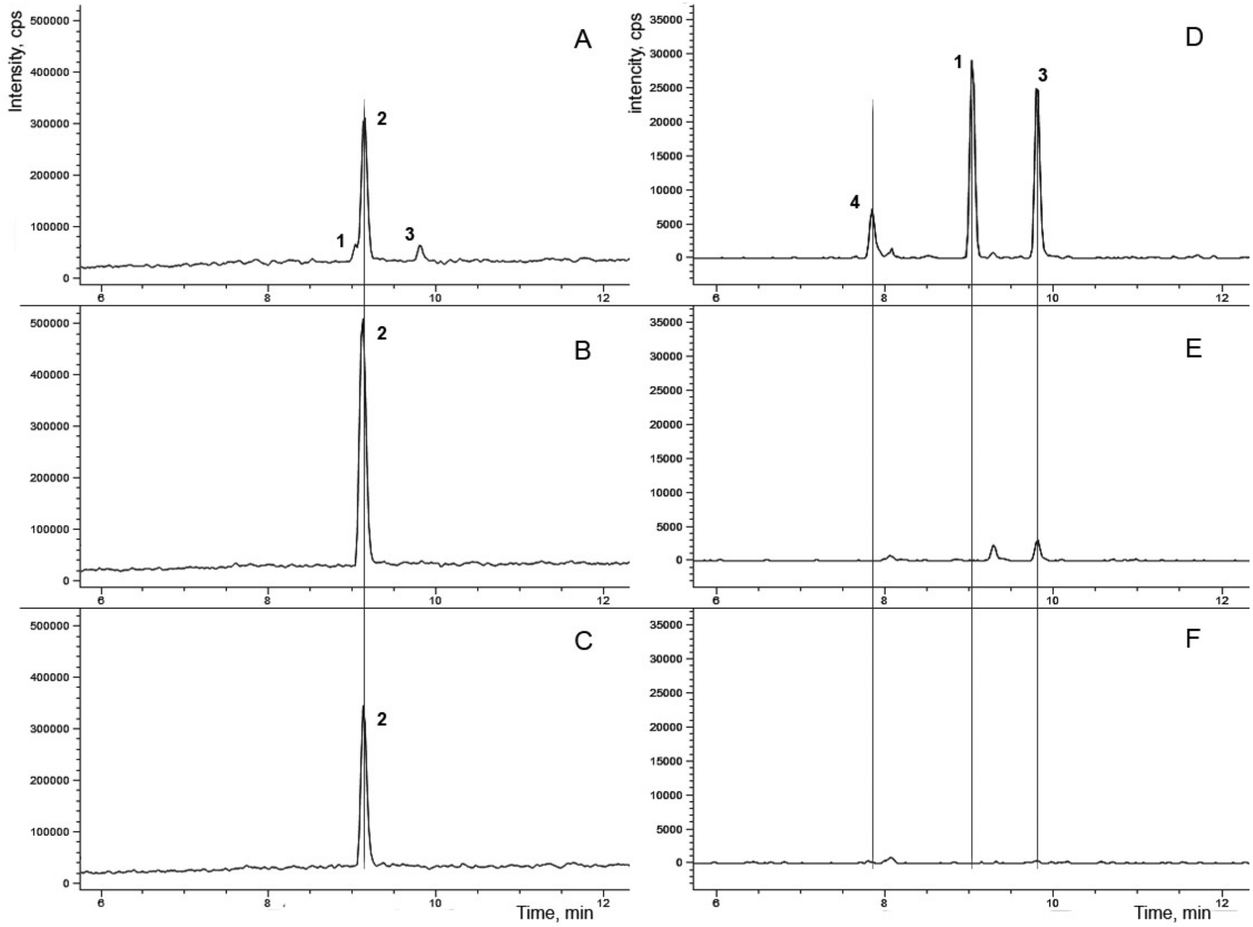

Figure S22. Fragments of HPLC-MS ion chromatograms of CYP3A4 baculosome samples. A) CYP3A4 baculosome preparation supplemented with vomilenine and NADPH; B) CYP3A4 baculosome preparation supplemented with vomilenine and lacking NADPH (control 1); C) CYP3A4 baculosome preparation inactivated by boiling and supplemented with vomilenine and NADPH (control 2); D–F) respective selective extraction of m/z 367 ion chromatogram. 1, 3 and 4, Ion signals m/z $[M+H]^+$ 367; 2, vomilenine (m/z $[M+H]^+$ 351).

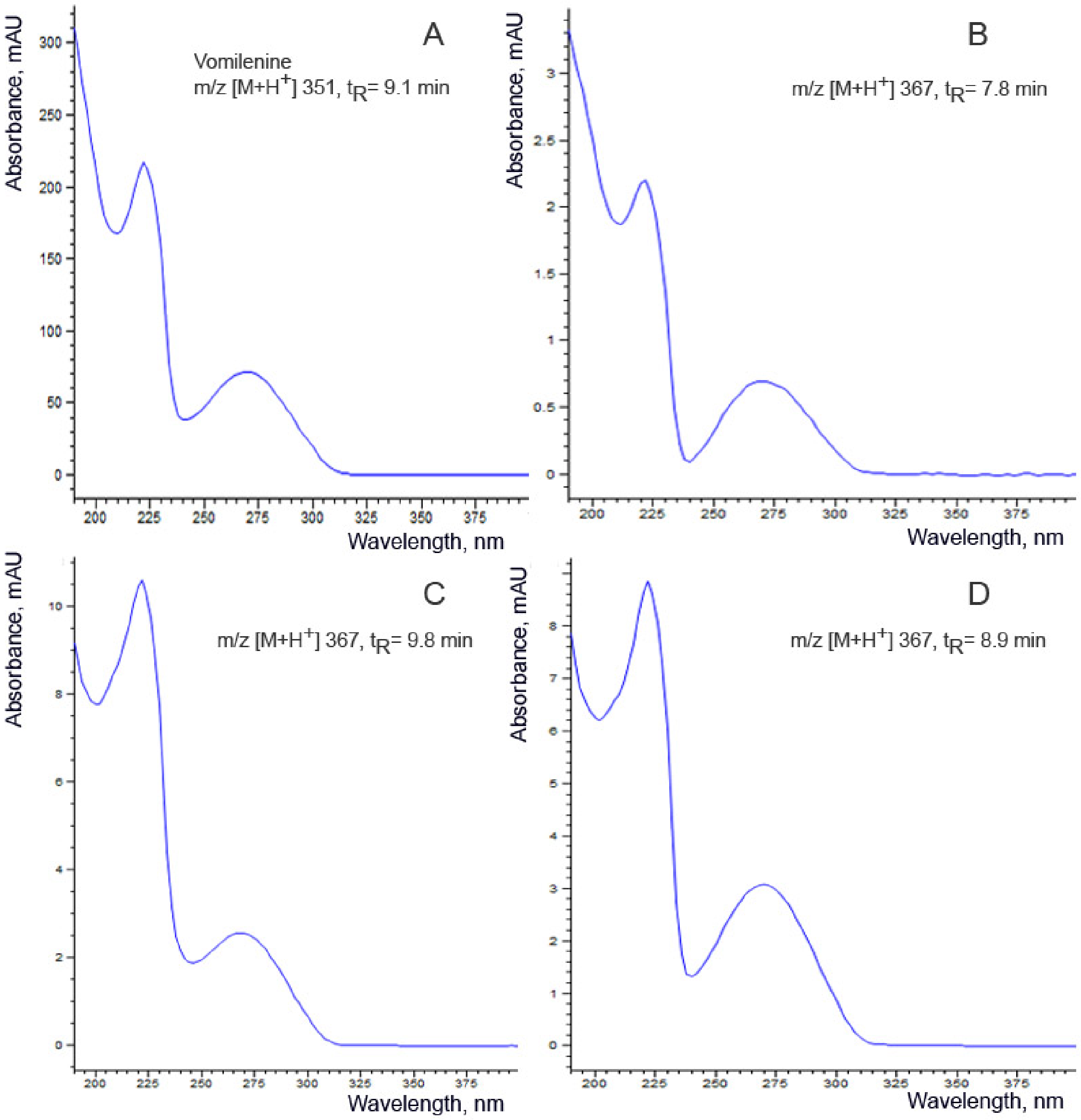

Figure S23. HPLC-UV spectra of vomilenine (A) and putative products of its oxidation by CYP3A4 (B–D).

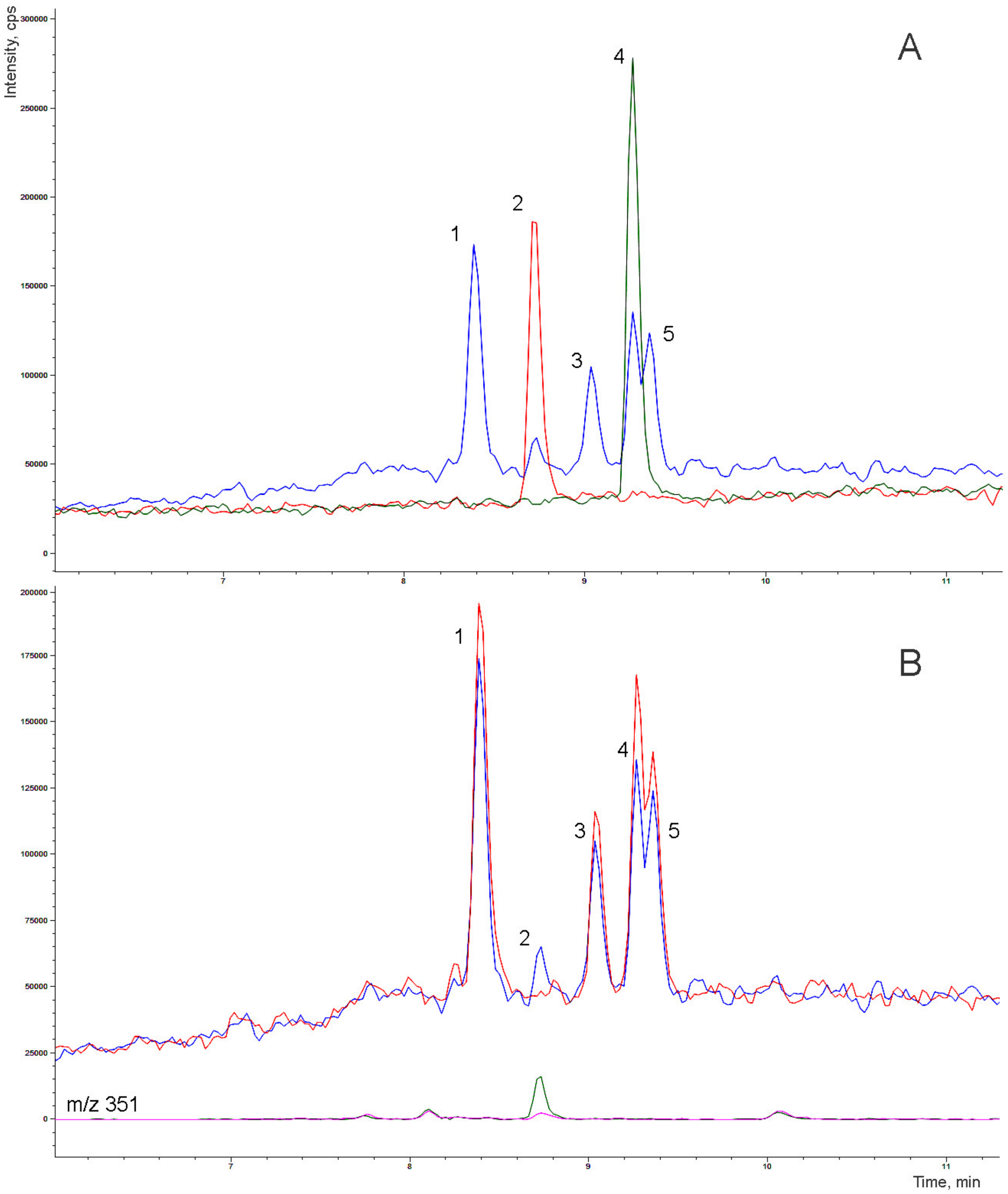

Figure S24. Fragments of HPLC-MS chromatograms of the extracts of *N. benthamiana* plants transiently expressing *CYP3A4* or *GFP* (control) and supplemented with vinorine. A) Total ion chromatogram of the extract of *N. benthamiana* plants transiently expressing *CYP3A4* (blue), standard sample of vinorine 19*R*, 20*R*-epoxide (red) and standard sample of vinorine (green); B) total ion chromatogram of the extracts of *N. benthamiana* plants

transiently expressing *CYP3A4* (blue) and *GFP* (red) with extracted ion m/z 351 chromatograms (green and purple, respectively). 1, 10-Deoxysarpagine (m/z $[M+H]^+$ 295); 2, vinorine 19*R*, 20*R*-epoxide (m/z $[M+H]^+$ 351); 3 and 5, vellosimine hemiacetal isomers (m/z $[M+H]^+$ 325); 4, vinorine (m/z $[M+H]^+$ 335).

S4. References

[S1] H. Warzecha, A. Frank, M. Peer, E. M. Gillam, F. P. Guengerich, M. Unger, *Plant Biotech. J.* **2007**, *5*, 185.

[S2] Y. V. Sheludko, I. M. Gerasymenko, H. Warzecha, *Biotech. J.* **2018**, *13*, e1700696.

[S3] I. Gerasymenko, Y. Sheludko, S. Frabel, A. Staniek, H. Warzecha, *Method. Enzymol.* **2019**, *617*, 413.

[S4] J. K. Yano, M. R. Wester, G. A. Schoch, K. J. Griffin, C. D. Stout, E. F. Johnson, *J. Biol. Chem.* **2004**, *279*, 38091.

[S5] C. Parage, E. Foureau, F. Kellner, V. Burlat, S. Mahroug, A. Lanoue, T. Duge de Bernonville, M. A. Londono, I. Carqueijeiro, A. Oudin, S. Besseau, N. Papon, G. Glevarec, L. Atehortua, N. Giglioli-Guivarc'h, B. St-Pierre, M. Clastre, S. E. O'Connor, V. Courdavault, *Plant Physiol.* **2016**, *172*, 1563.

[S6] K. Miettinen, L. Dong, N. Navrot, T. Schneider, V. Burlat, J. Pollier, L. Woittiez, S. van der Krol, R. Lugan, T. Ilc, R. Verpoorte, K. M. Oksman-Caldentey, E. Martinoia, H. Bouwmeester, A. Goossens, J. Memelink, D. Werck-Reichhart, *Nat. Commun.* **2014**, *5*, 3606.
